# Supplementary material for: Dual interference with host neuropeptide signaling allows parasitoid wasp to hijack host sugar metabolism
Source: EMBO J. 2025 Nov 26;45(6):2030–50. doi: 10.1038/s44318-025-00636-5 (PMC12992715; doi:10.1038/s44318-025-00636-5)
Supplement: Supplementary file 1 — Appendix [file 44318_2025_636_MOESM1_ESM.pdf]

Appendix for
Dual interference with host neuropeptide signaling allows parasitoid wasp to hijack host sugar metabolism

Zhi-Zhi Wang[1,2,3,4,5] Ruo-Fei Ma[1,2,3,5], Li-Cheng Gu[1,2,3], Li-Zhi Wang[1,2,3], Ting Chen[1,2,3], Pei Yang[1,2,3], Jia-Ni Zou[1,2,3], Jiang-Yan Zhu[1,2,3], Zhi-Wei Wu[1,2,3], Yue-Nan Zhou[1,2,3], Min Shi[2,3], Xing-Xing Shen[1,2,3], Jian-Hua Huang[1,2,3], Xue-Xin Chen[1,2,3,4]*

[1]Institute of Insect Sciences, College of Agriculture and Biotechnology, Zhejiang University, Hangzhou, China

[2]Ministry of Agriculture and Rural Affairs Key Lab of Molecular Biology of Crop Pathogens and Insect Pests, Zhejiang University, Hangzhou, China

[3]Key Laboratory of Biology of Crop Pathogens and Insects of Zhejiang Province, Zhejiang University, Hangzhou, China

[4]State Key Lab of Rice Biology and Breeding, Zhejiang University, Hangzhou, China

[5]These authors contributed equally
* Corresponding author: Xue-Xin Chen
Email: xxchen@zju.edu.cn

This PDF file includes:

Appendix Figures S1 to S10
Appendix Tables S1 to S5

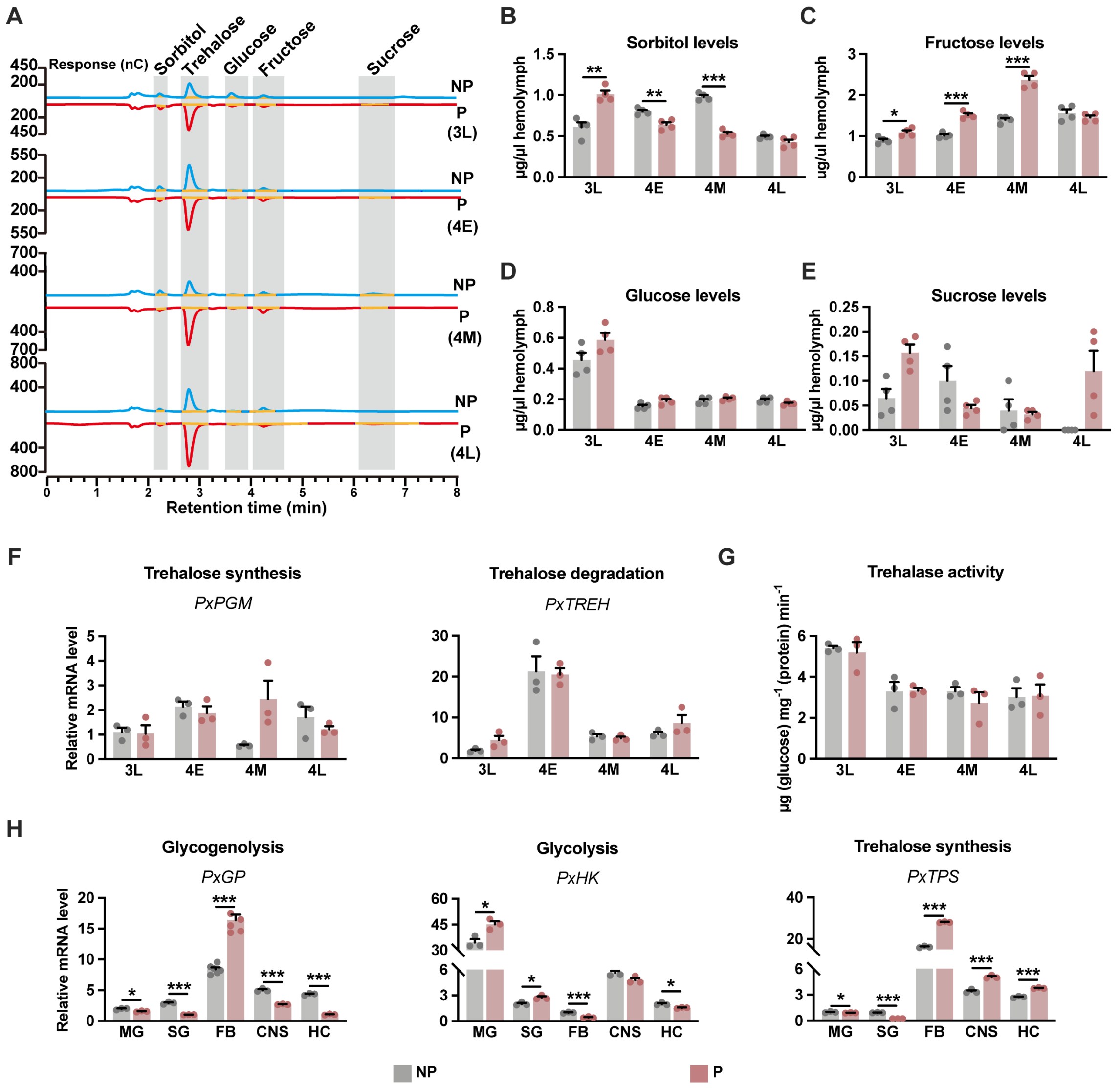

Appendix Figure S1. *C. vestalis* parasitism induces disorders of carbohydrate metabolism in the host.

(**A**) Representative HPLC-derived chromatogram of hemolymph aqueous extracts acquired at the indicated times for non-parasitized (blue, NP) or *C. vestalis*-parasitized (red, inverted for clarity, P) host larvae. Grey vertical bars highlight the peaks of five carbohydrates. The retention times of the standards are as follows: sorbitol (2.35 min), trehalose (2.79 min), glucose (3.61 min), fructose (4.19 min) and sucrose (6.25 min). (**B-E**) Sorbitol (B), Fructose (C), Glucose (D) and Sucrose (E) levels in hemolymph from non-parasitized and *C. vestalis*-parasitized host larvae at different instars (n = 4 replicates of eight moths).
(**F**) Relative mRNA levels of *PxPGM* (left) and *PxTREH* (right) from non-parasitized and *C. vestalis*-parasitized host larvae (whole body) at different instars (n = 3). (**G**) Trehalase activity of non-parasitized and *C. vestalis*-parasitized host larvae at different instars (n = 3). (**H**) Relative mRNA levels of *PxGP* (left), *PxHK* (middle) and *PxTPS* (right) in different tissues of non-parasitized and *C. vestalis*-parasitized host larvae at middle 4th instar (n = 3~6). 3L, late 3rd-instar; 4E, early 4th-instar; 4M, middle 4th-instar; 4L, late 4th-instar. MG, midgut; SG, silk gland; FB, fat body; CNS, central nervous system; HC, hemocytes. Data are represented as mean ± SEM. * $p < 0.05$, ** $p < 0.01$, *** $p < 0.005$; not shown, no significance. Two-tailed unpaired Student's *t*-test.

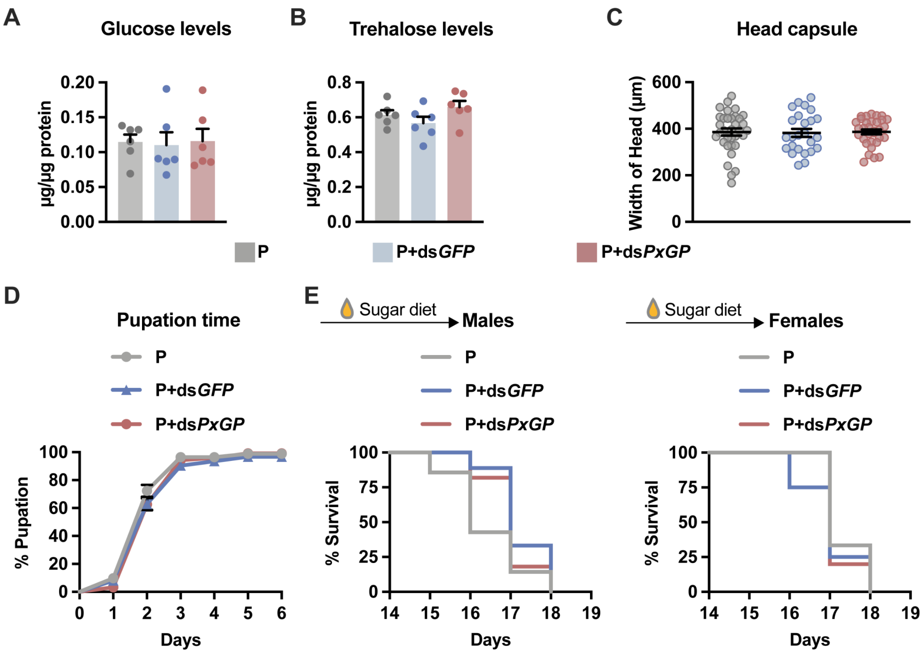

Appendix Figure S2. Glucose levels, trehalose levels, head capsule width and pupation time of offspring wasps were not affected by the lower trehalose concentration in parasitized host hemolymph

(**A-D**) Glucose content (**A**, n = 6), trehalose content (**B**, n = 6), head capsule width (**C**, n = 34 for grey, n = 25 for blue, n = 32 for red), and pupation time (**D**, n = 4) of *C. vestalis* emerged from ds*PxGFP*, ds*PxGP* or non-treated host *P. xylostella* larvae. (**E**) Survival curves of *C. vestalis* males (left, n = 28 for grey, n = 27 for blue, n = 22 for red) and females (right, n = 21 for grey, n = 32 for blue, n = 25 for red) after eclosion from ds*PxGFP*, ds*PxGP* or non-treated host *P. xylostella* larvae. Males and females from each experimental batch were reared on a honey diet. Experimental design of (A-E) is the same as in Fig. 2A. Data are represented as mean ± SEM. ns, no significance. One-way ANOVA and Tukey's multiple comparison tests, except for (D and E) (Kaplan-Meier log-rank test). p value not shown as it was not significant.

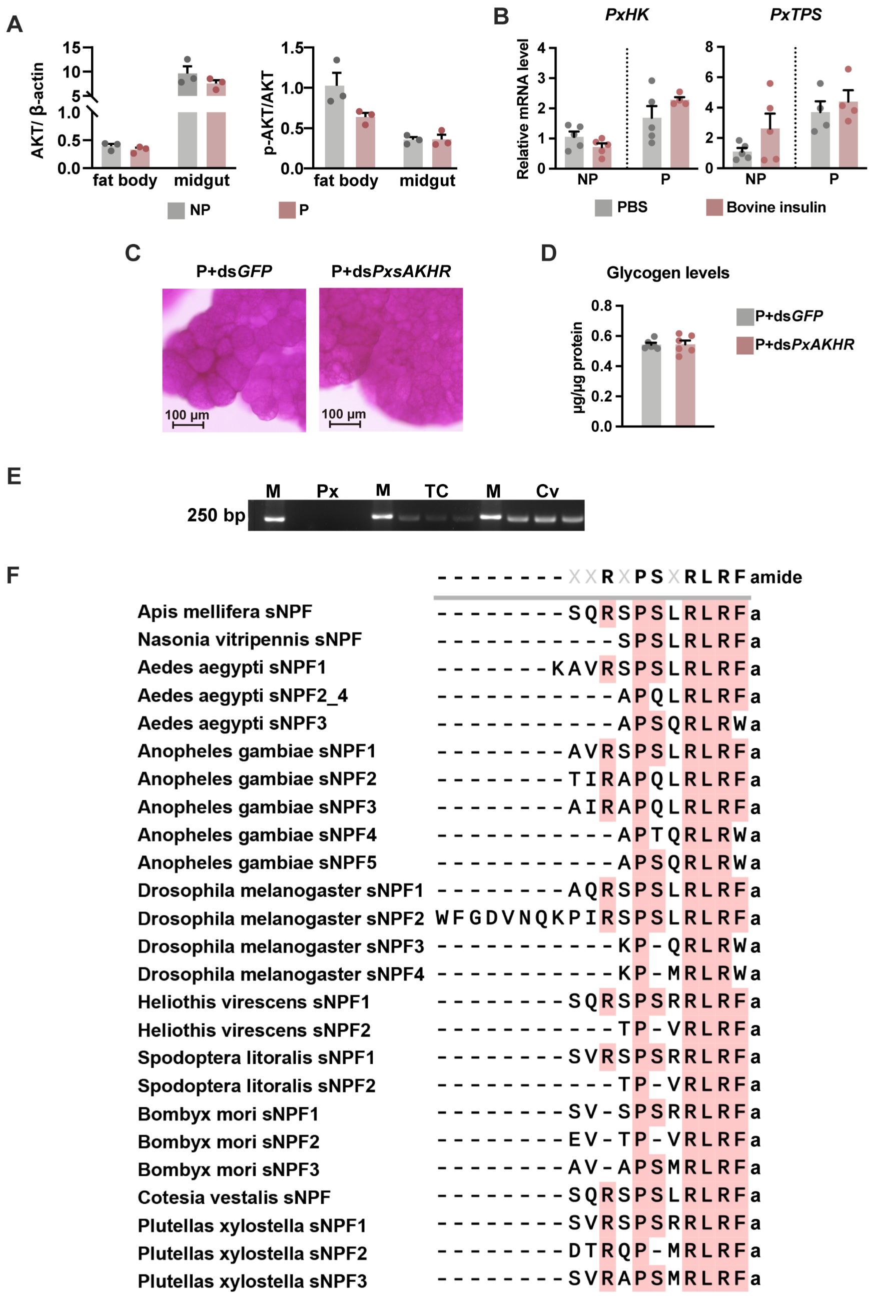

Appendix Figure S3. Effects of insulin and AKH signalling on trehalose metabolism and analysis of insect sNPF peptides.

(**A**) The relative protein levels of AKT/β-actin (left) and pAKT/AKT (right) were measured by Image J. (**B**) Relative mRNA levels of *PxHK* and *PxTPS* from 4M instar hosts (whole body) treated with bovine insulin (n=4-5). (**C** and **D**) PAS staining of the fat body (**C**) and its glycogen content (**D**, n = 6) after *PxAKHR* knockdown. Scale bars, 100 μm. (**E**) PCR detection of CvsNPFs in teratocytes. The wasp larvae and host hemolymph were used as positive control and negative control, respectively. M, DNA marker, Px, hemolymph of *P. xylostella* larvae (4L), TC, teratocytes, Cv, 1-day old *C. vestalis* adult. (**F**) Multiple alignment of predicted CvsNPF and PxsNPF peptides with sNPF from other insects. The sequence (xPxLRLRFamide) indicates the conserved C-terminal motif. Identical residues are highlighted in pink. Data are represented as mean ± SEM. Two-tailed unpaired Student's *t*-test, p value not shown as it was not significant.

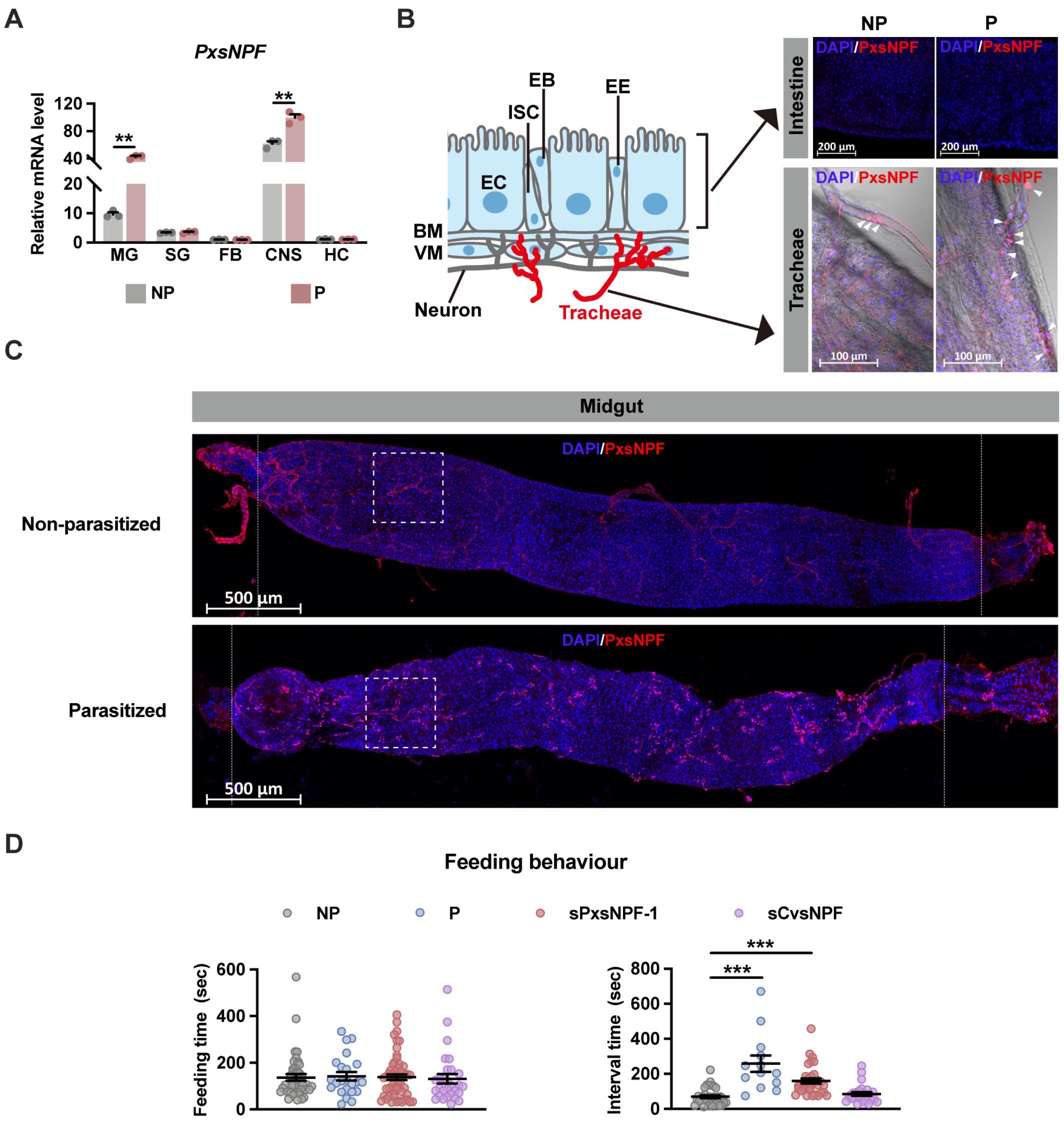

Appendix Figure S4. PxsNPF is mainly expressed in the CNS and midgut-trachea of the host as an appetite-suppressing hormone

(**A**) Relative mRNA levels of *PxsNPF* in different tissues of non-parasitized and *C. vestalis*-parasitized host larvae at late 3rd instar (n = 3). (**B**) Left: schematic diagram of the general composition and cell types of the midgut. EC, enterocyte; ISC, intestinal stem cell; EB, enteroblast; EE, enteroendocrine cell; BM, basement membrane; VM, visceral muscle. Right: PxsNPF (red) is expressed in the tracheae of the midgut (white arrowheads). Nuclei were labelled by DAPI (blue). NP: non-parasitized hosts, P: parasitized hosts. Scale bars, 200 μm. (**C**) Immunostaining of PxsNPF prepropeptide (red) in the whole midgut of non-parasitized and parasitized *P. xylostella* larvae at late 3rd-instar. White dashed boxes indicate areas magnified in Fig. 5E. Nuclei were labelled by DAPI (blue). Scale bar: 500 μm. (**D**) Feeding time (left, n = 40 for grey, n = 23 for blue, n = 54 for red, n = 29 for purple) and interval time (right, n = 35 for grey, n = 13 for blue, n = 33 for red, n = 25 for purple) of non-parasitized, *C. vestalis*-parasitized, and sPxsNPF-1-injected and sCvsNPF-injected *P. xylostella* larvae at middle 4th-instar during feeding behaviour. Data are represented as mean ± SEM. $^{**}p < 0.01$, $^{***}p < 0.005$; not shown, no significance. Two-tailed unpaired Student's *t*-test, except for (**D**) (One-way ANOVA and Tukey's multiple comparison tests).

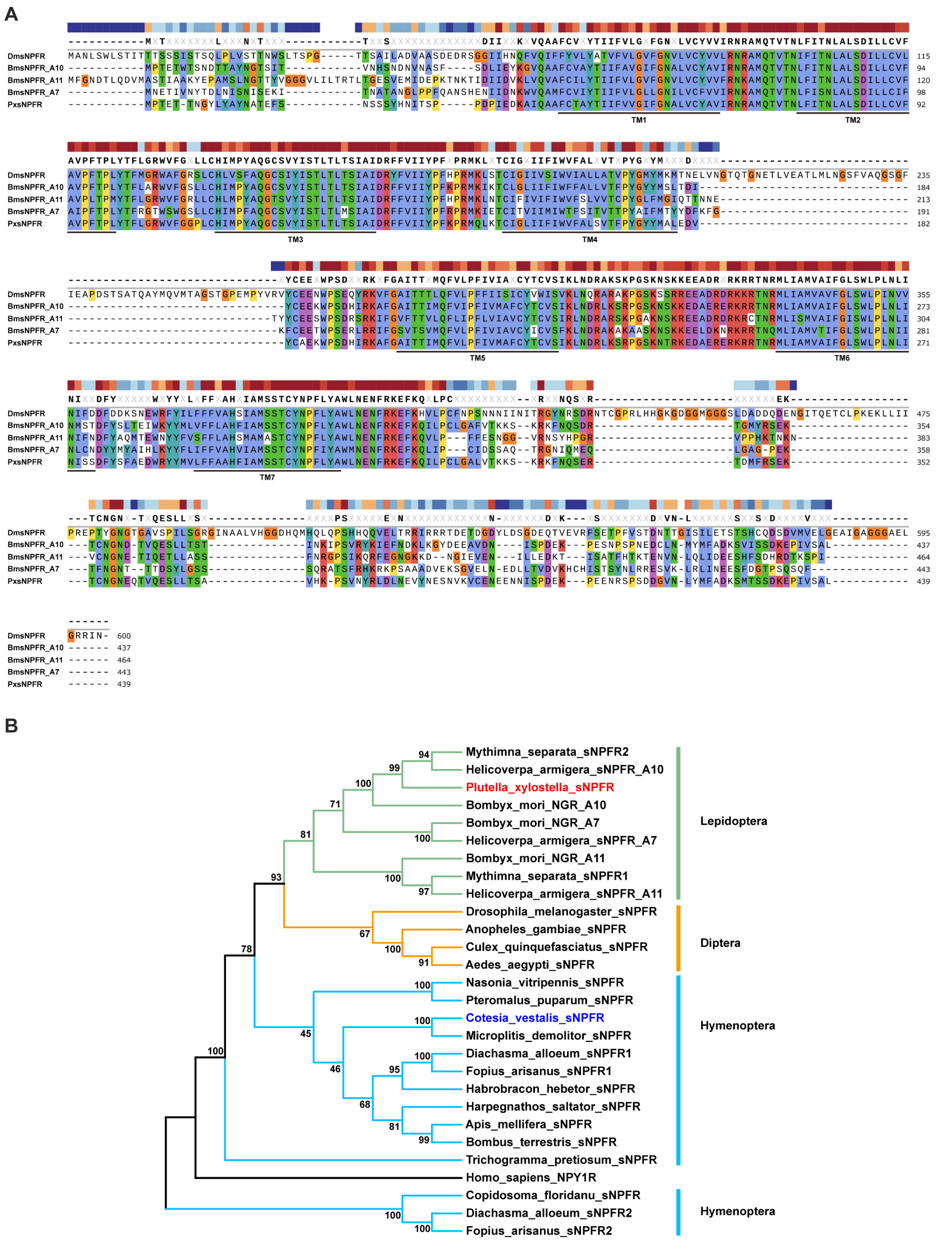

Appendix Figure S5. Sequence and phylogenetic analysis of *PxsNPFR*.

(**A**) Multiple alignment of predicted PxsNPFR with sNPFRs from other insects. The underlined amino acids indicate seven typical transmembrane domains. (**B**) Phylogenetic tree analysis of PxsNPFR with sNPFRs homologs in other insects and *H. sapiens*. The percent bootstrap support values are indicated by the number of branches. The red labels are sNPFR from *P. xylostella* and the blue labels are sNPFR from *C. vestalis*. Homologous sequences were obtained from NCBI (https://www.ncbi.nlm.nih.gov/), including *P. xylostella* (XP_011565707.1), *Mythimna separata* (WEU67074.1, WEU67075.1), *Helicoverpa armigera* (WGD18953.1, WGD18957.1, WGD18956.1), *Bombyx mori* (BAG68406.1, NP_001127707.1, NP_001127708.1), *D. melanogaster* (NP_001262086.1), *Anopheles gambiae* (ABD96049.1), *Culex quinquefasciatus* (AVR59280.1), *Aedes aegypti* (XP_021705044.1), *Habrobracon hebetor* (Hheb089310.1), *Harpegnathos saltator* (EFN78163.1), *Apis mellifera* (XP_006561748.1), *Bombus terrestris* (XP_020719990.1), *Nasonia vitripennis* (NV15762-RA), *Pteromalus puparum* (PPU01679-RA), *Homo sapiens* (AAA59920.1).

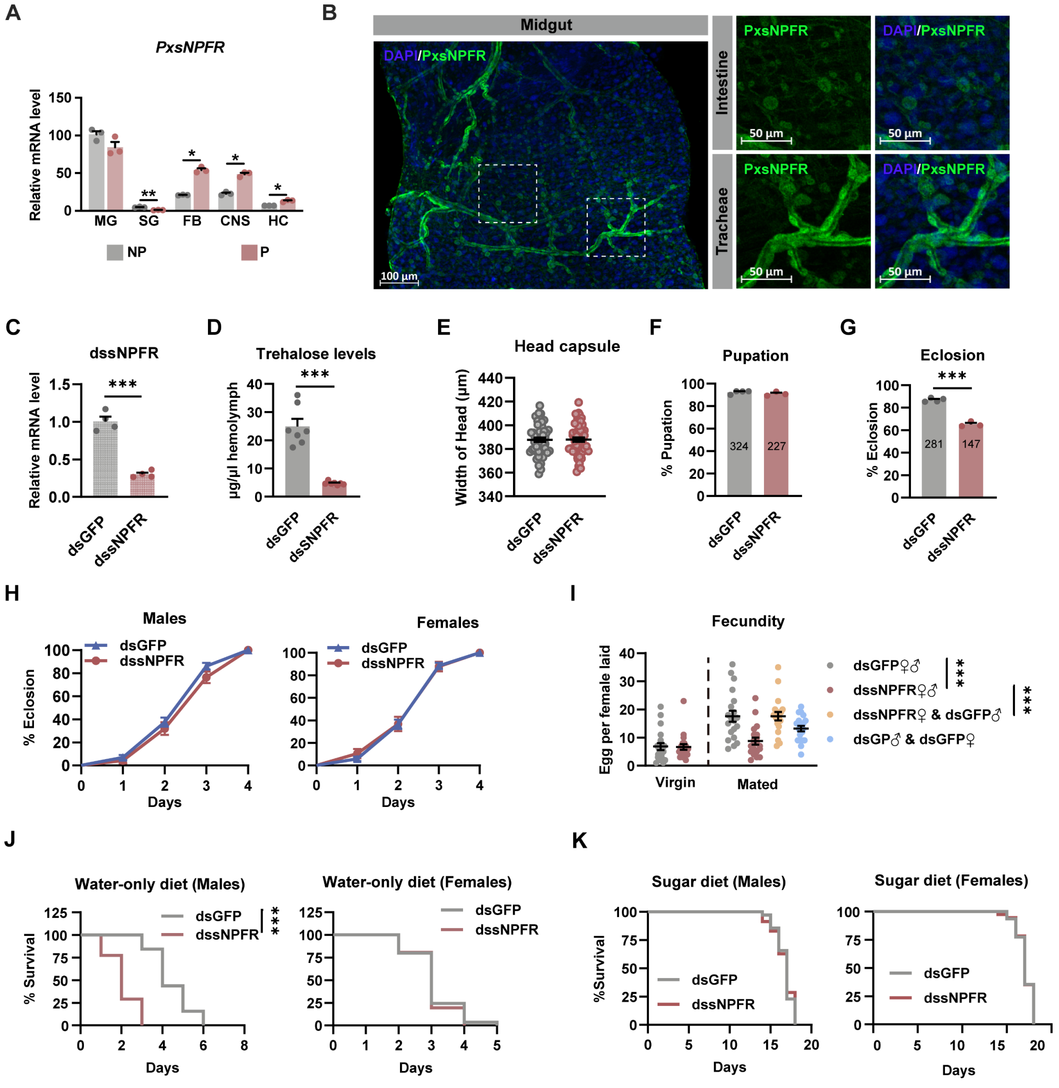

Appendix Figure S6. *PxsNPFR* expression profile and the effect of ds*PxsNPFR* on wasp fitness.

(**A**) Relative mRNA levels of *PxNPFR* in different tissues of non-parasitized and *C. vestalis*-parasitized host larvae at middle 4th instar (n = 3). (**B**) PxsNPFR immunostaining (green) in the midgut of non-parasitized *P. xylostella* larvae at late 3rd-instar. Nuclei were labelled by DAPI (blue). White dashed boxes indicate areas magnified in the far-right panels. Scale bar: 100 µm (left) and 50 µm (right). (**C**) RNAi efficiency of ds*PxsNPFR* in parasitized host larvae (whole body) at 3L instar (n = 4). ds*PxGFP* was used as the control. (**D**) Circulating trehalose concentration from C. vestalis parasitized host larvae treated with dsPxGFP or dsPxsNPFR (n=7). (**E**) Head capsule width of wasp larvae from ds*PxsNPFR* treated host larvae (n=50). (**F-G**) pupation rate (E, n = 3-4) and eclosion rate (F, n = 3) of wasp offspring. dsPxsNPFR. The total number of wasps for each treatment in (**F** and **G**) was shown on each bar graph. (**H**) Eclosion time of *C. vestalis* males (n = 144 for blue and n = 81 for red) and females n = 137 for blue, n = 66 for red). (**I**) Number of eggs laid per hour by each virgin (left) or mated (right) female offspring. Adult parasitoid wasps (both mated and virgin) used for parasitization were fed a sugar diet for approximately two days prior to the experiment. (**J**) Survival curves of *C. vestalis* males (n = 32 for grey, n = 31for red) and females (n = 30 for grey, n = 31 for red) fed with water. (**K**) Survival curves of *C. vestalis* males (n = 32 for grey, n = 31for red) and females (n = 30 for grey, n = 31 for red) fed with sugar diet. Data are represented as mean ± SEM. Two-tailed unpaired Student's *t*-test for (**A**, **C**-**G**). One-way ANOVA and Tukey's multiple comparison tests for (**H**, **I**), and Kaplan-Meier log-rank test for (**J**, **K**). *** p $<$ 0.01, not shown p > 0.05.

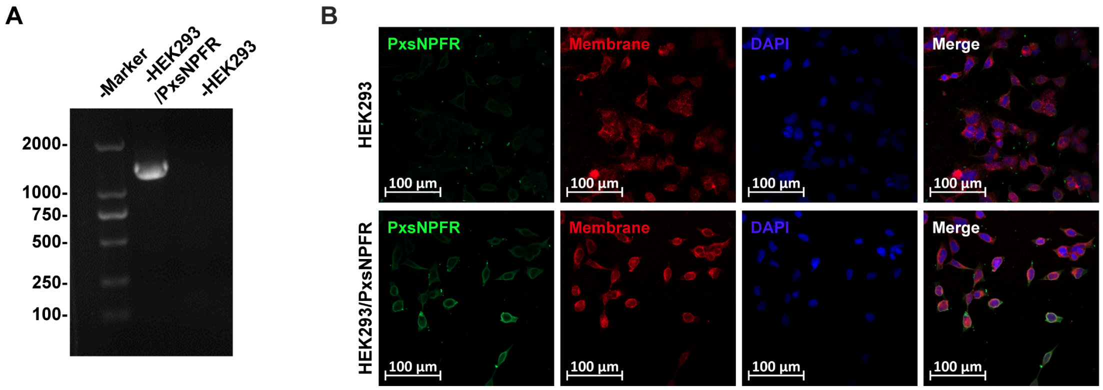

Appendix Figure S7. Localization of PxsNPFR in host midgut and HEK293/PxsNPFR cell lines. (A) The detection of *PxsNPFR* in HEK293/PxsNPFR cell lines. HEK293 cell lines without PxsNPFR transfection served as the control group. (B) PxsNPFR immunostaining (green) was localized on the membrane in HEK293/PxsNPFR cells but not in HEK293 control cells. Membranes were labelled by Plasma Membrane Stains (red). Nuclei were labelled by DAPI (blue). Scale bars: 100 µm.

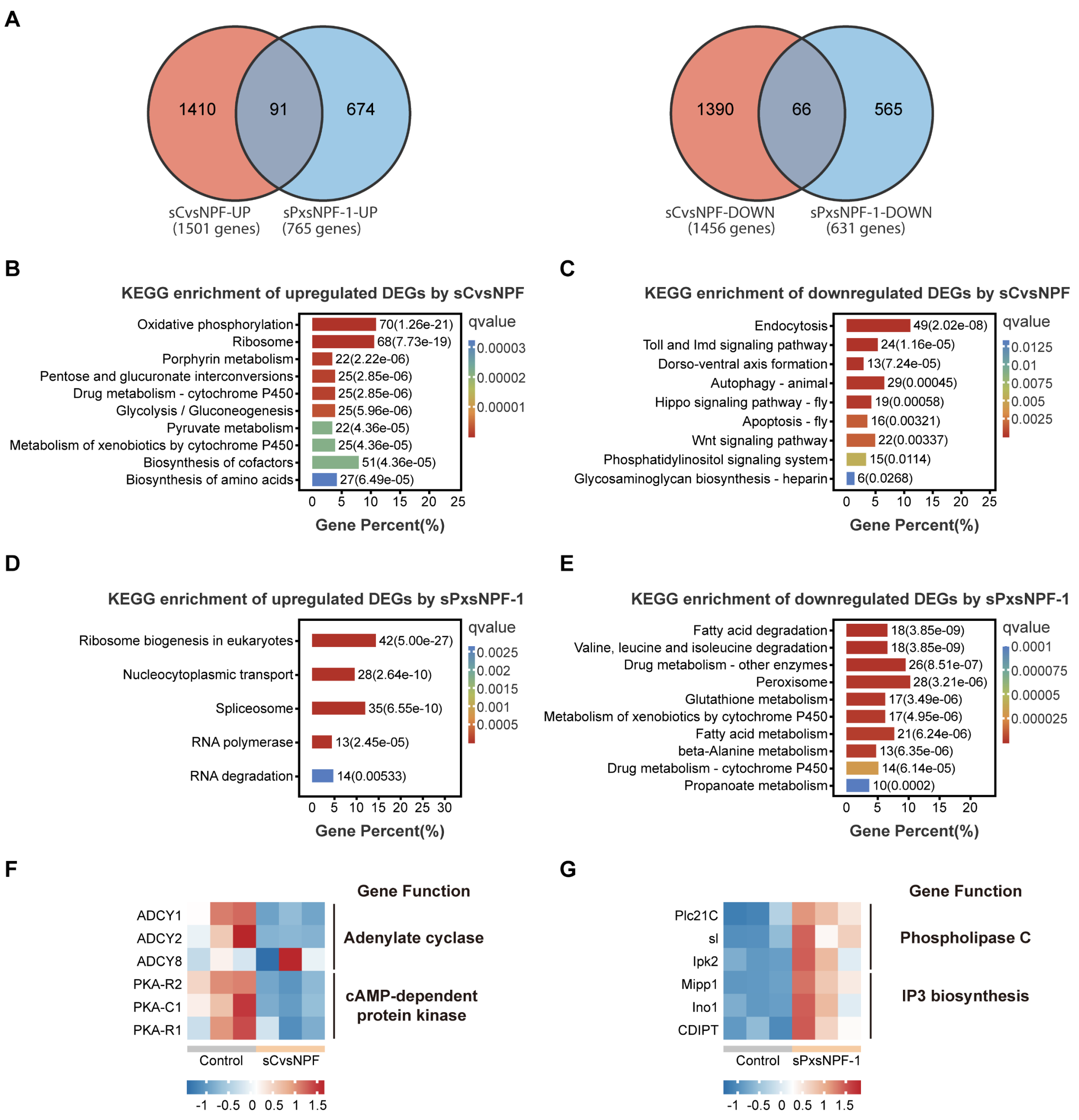

Appendix Figure S8. Downstream signalling pathways enriched by sCvsNPF and sPxsNPF-1 treatments exhibit transcriptome divergence.

(**A**) Venn diagram showing differentially expressed transcripts in the sCvsNPF vs. sPxsNPF-1 comparisons. The number of transcripts individually or co-enriched (p value < 0.05) is indicated for each overlap. The DEGs in these different categories are listed in Datasets S1 and S2. (**B**-**E**) KEGG pathway enrichment of DEGs identified from the comparisons of CK vs. sCvsNPF-injected *P. xylostella* larval fat body (**B** and **C**) and CK vs. sPxsNPF-1-injected *P. xylostella* larval midgut (**D** and **E**) at the middle 4th instar. (**F** and **G**) RNA-seq analysis of the fat body of *P. xylostella* injected with sCvsNPF or not (**F**), and the midgut of *P. xylostella* injected with sPxsNPF-1 or not (**G**). A list of differentially expressed PKA-related genes and PLC-related genes was shown. Red and blue colours respectively represent high and low expression levels based on the lg (FPKM) values. ADCY1, XM_011550110.1; ADCY2, XM_011552086.1; ADCY8, XM_011552464.1; PKA-R2, XM_011553353.1; PKA-C1, XM_011555032.1; PKA-R1, XM_011566500.1; Plc21C, XM_011550872.1; sl, XM_011550838.1; Ipk2, XM_011555251.1; Mipp1, XM_011559789.1; Ino1, XM_048624407.1; CDIPT, XM_011557524.3.

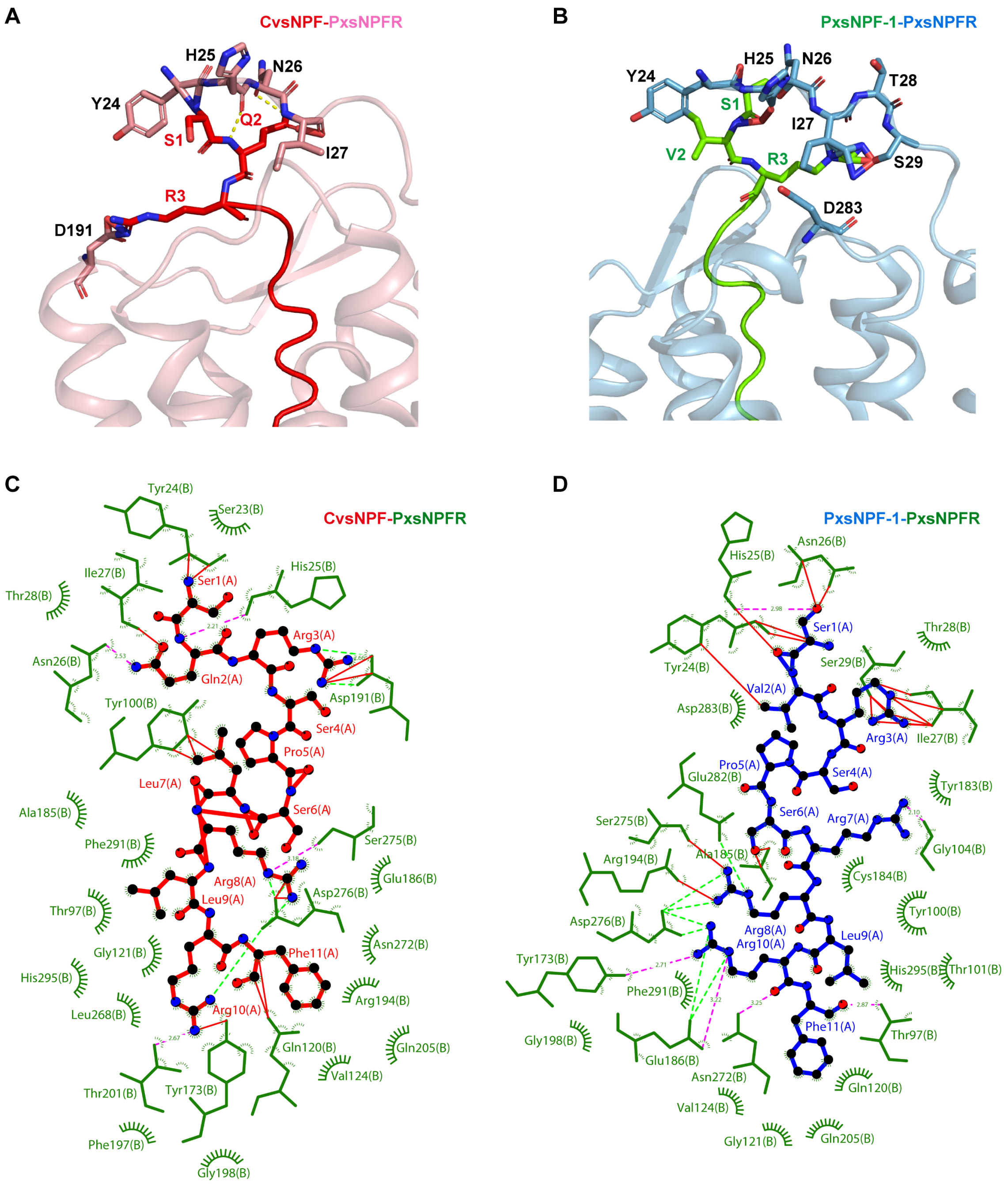

Appendix Figure S9. Interaction patterns comparison between CvsNPF-PxsNPFR and PxsNPF-1-PxsNPFR.

(**A** and **B**) Interactions between PxsNPFR and the peptide residues S1-R3 shows that residue Q2 in CvsNPF binds with I27 and N26 in the C-terminal of the receptor, whereas V2 in PxsNPF-1 interacts with Y24 of the receptor. The hydrogen bonds are displayed as yellow dashed lines.

(**C** and **D**) Schematic representation of CvsNPF–PxsNPFR (**C**) and PxsNPF-1–PxsNPFR (**D**) interactions, analyzed using the LigPlot+ program is shown. The position of residue R3 differed between CvsNPF and PxsNPF-1, with R3 from CvsNPF engaging with D191 of PxsNPFR and R3 from PxsNPF-1 interacting with S29 and I27 of the extracellular C-terminal region of the receptor. The hydrogen bonds and the salt bridges are shown in magenta and green dashed lines, respectively.

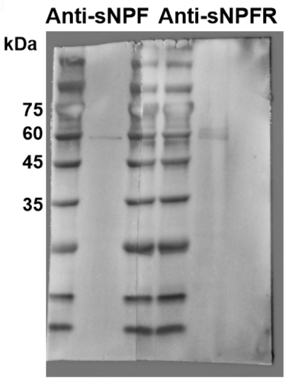

Appendix Figure S10. Western blotting analysis of midguts of 4M instar host larvae against anti-sNPF (left, 1:500) and anti-sNPFR (right, 1:500) antibodies. The HRP Goat Anti-Rabbit IgG (ABclonal, 1: 2500 dilution) was used as the secondary antibody. As neuropeptides are usually proteolytically cleaved during secretion, the detected band of anti-sNPF is a partially processed propeptide prior to cleavage. Similarly, the band detected by anti-PxsNPFR is larger than expected size, may also represent an immature form of PxsNPFR or a glycosylated form. Interestingly, both bands were found at a similar molecular weight.

Appendix Table S1. The area time of carbohydrate standard samples.

Standard sample	Area time (min)
Sorbitol	2.35
Trehalose	2.79
Arabinose	3.00
Maltose	3.12
Mannose	3.41
Glucose	3.61
Xylose	3.87
Fructose	4.19
Lactose	5.54
Sucrose	6.25

Appendix Table S2. GenBank Accession Numbers of insulin-PI3K/AKT signalling-related genes.

Gene	GenBank Accession Number
ILP1	XM_011554348.3
ILP3	XM_011565709.3
InR	XM_011569614.1
PI3K	XM_011559348.1
AKT	XM_011550518.1
PDK	XM_011551465.1

Appendix Table 3. LC-MS analysis of the SDS-PAGE bands detected by the sNPF and sNPFR antibodies

Description	Coverage [%]	# Unique Peptides	MW [kDa]	Confidence	XCorr
**Cv-sNPF**	85	8	12.5		
Annotated Sequence	Modifications	# PSMs	Positions		
[R].SDPMIPKGNLLTR.[S]		3	Cv-sNPF [85-97]	High	1.83
[R].NSLEGLRSSMSVDSPYEHLMIR.[K]		13	Cv-sNPF [49-70]	High	2.97
[R].SYRCAIVLFFILGAALAAENYLDYGEENADR.[N]	1xCarbamidomethyl [C4]	1	Cv-sNPF [3-33]	High	2.52
[R].CAIVLFFILGAALAAENYLDYGEENADRNIENLR.[E]	1xCarbamidomethyl [C1]	4	Cv-sNPF [6-39]	High	3
[R].RNSLEGLR.[S]		6	Cv-sNPF [48-55]	High	2.03
[K].GNLLTRSASNPASFEDN.[-]		1	Cv-sNPF [92-108]	High	1.99
[K].SQRSPSLR.[L]		4	Cv-sNPF [72-79]	High	1.3
[R].RNSLEGLRSSMSVDSPYEHLMI.[R]		1	Cv-sNPF [48-69]	High	1.28
**Px-sNPF**	34	4	19.6		
Annotated Sequence	Modifications	# PSMs	Positions		
[R].RSDPDMPPQAPLDEMEELLSLR.[D]		1	Px-sNPF [85-106]	High	0.92
[R].SDPDMPPQAPLDEMEELLSLR.[D]	2xOxidation [M5; M14]	1	Px-sNPF [86-106]	High	1.07
[R].SAETAVPHVFPQEEQDRSVR.[A]		1	Px-sNPF [120-139]	High	1.32
[R].SDNNMFLLPYESALPKDVK.[A]	1xOxidation [M5]	1	Px-sNPF [151-169]	High	1.28
**PxsNPFR**	8	1	50.2		
Annotated Sequence	Modifications	# PSMs	Positions		
[R].MLIAMVAIFGLSWLPLNLINISSDFYSFAEDWR.[Y]	1xOxidation [M5]	1	PxsNPFR [253-285]	High	0.86

PSM: Peptide Spectrum Match

Appendix Table S4 Primer used in this study.

Gene	GenBank Accession Number	Forward (5'-3')	Reverse (5'-3')	Purpose
PxGP	XM_011561323.1	AATACCAAGAAACTCCCAAAT	AGGACTATGTGCTATCTCGCTC	Cloning
PxPGM	XM_011550792.1	AATACGCAAAAATGCCAGG	GTGGCAAACCTAACGAAGCAAG	Cloning
PxHK	XM_011557932.1	CGCTCCTTTCACTCCTCCAC	CGTACAACGCGATTCCGTGAG	Cloning
PxTPS	XM_011554961.1	ACACCCCACCGTTGTTTCTGC	ACCTCCCACTAAACTCCCATC	Cloning
PxTREH	XM_011557077.1	CCCCAGTTCCTGTCCCGCAT	GAAGGTTGTGGGGATGCCG	Cloning
PxsNPF	XM_011567405.3	CGAGGATTTTTCAGATTTCATTACC	ATTAGTGCTTGTGTTTCGGTTTG	Cloning
PxsNPFR	XM_038106752.2	ATGCCGACGGAAACTACCAAAT	CTAGAGCGCGGACACGATGGGCT	Cloning
PxAKH_I	KJ783476.1	GGGAAACTCTTCTAGTCAGC	CGGTTGTCAAAGACGCGTAG	Cloning
PxAKH_II	KJ783477.1	TCTGTTGAGAGTGTAGTGAA	GCACCTAATCTTGTCCTAGA	Cloning
PxAKHR	XM_048628030.1	ATGGACATCGACGAAAAAGTG	AACCAAATAACTATAAACAGCACTA	Cloning
CvsNPF	PP349728.1	ATGAGGAGTTACAGATGCGCT	TCAATTGTCCTCAAAAGATG	Cloning
PxGP		CGTTGATACACCGCAAGGGAA	ACTGCCTGGATGTAGTCGCCG	qPCR
PxPGM		CTCGTCAAGGAGGTTGTGGAT	TTGATGCCGAAATCGTTGTCT	qPCR
PxHK		CAATAGCCAGACGAGGGGACG	ATGCCCCCCATTCTGTGTTT	qPCR
PxTPS		TAACTGGATTAGACAGACCGCT	CAGGCAGTAGTCTGTGATGTGG	qPCR
PxTREH		ACTCAACGGCACCAACAAGGG	ACGTCCTCGTGCCACAACACC	qPCR
PxsNPF		GGCGGCTGAGATTTGGAC	CGACTCGTAGGGCAGTAGGA	qPCR
PxsNPFR		CTTGCTAACGGATCTTACAACTACA	AGGGGTATATATTTGAATGTTGTCG	qPCR
PxAKH_I		TTGGTGATCGTCAGTGAG	CTGGCATAGAAGAAGTCTCT	qPCR
PxAKH_II		ATGTTGGTGTTGGCAGAC	GCTTCGGTTTCAATGAGTTT	qPCR
PxAKHR		TGGACCAGGAGTCGGTGATG	TTTGGGGTGGATTGCGT	qPCR
PxInR		TGAGGTGTGTGGAAGAAC	TGAGGTGTGTGGAAGAAC	qPCR
Pxβ-tubulin		GACGCATGTCCATGAAGGAG	CCAATGCAAGAAAGCCTTGC	qPCR

Pxβ-actin	TGGCACCACACCTTCTAC	CATGATCTGGGTCATCTTCT	qPCR
PxGP	TAATACGACTCACTATAGGCGCCTTACA ACTCAACGAT	TAATACGACTCACTATAGGCGCAGA TCAGGTCAGAGAG	RNAi
PxHK	TAATACGACTCACTATAGGTGCCTGCTA TGTAGAAAAGAT	TAATACGACTCACTATAGGGGCTCG TCCATCTTGTTGA	RNAi
PxsNPF	TAATACGACTCACTATAGGGCTCTCACA GTATGACTCAGTGG	TAATACGACTCACTATAGGTTGCTC CTCCTGCGGGAAGA	RNAi
PxsNPFR	TAATACGACTCACTATAGGGCAAACCGT CACTAATCTCTTCA	TAATACGACTCACTATAGGTCTCCG CATCTTCTTTTCTCGTA	RNAi
PxAKHR	TAATACGACTCACTATAGGCTTCGTGCT CATCTGTATT	TAATACGACTCACTATAGGGGCTGT TAGGATTCGTCTT	RNAi
GFP	TAATACGACTCACTATAGGCAGTGCTTC AGCCGCTACCC	TAATACGACTCACTATAGGCTTCTC GTTGGGGTCTTTGCT	RNAi
PxsNPFR	CGCGGATCCATGCCGACGGAAACTACAA ATGGAT	CCGCTCGAGCTAGAGCGCGGACACG ATGGGCTCC	Vector construction

Appendix Table S5. Exact P Values for the Figures

Figure 1

	NP vs. P	Significant	Pvalue
Figure 1B	3L	***	0.000033
	4E	***	0.000084
	4M	***	0.00007
	4L	***	0.000006
Figure 1D（GP）	3L	ns	0.32932
	4E	**	0.004663
	4M	**	0.001313
	4L	*	0.039465
Figure 1D（HK）	3L	**	0.002374
	4E	ns	0.830517
	4M	**	0.001676
	4L	ns	0.01887
Figure 1D（TPS）	3L	ns	0.702382
	4E	ns	0.915172
	4M	*	0.016766
	4L	*	0.011974
Figure 1E	3L	**	0.001447
	4E	ns	0.86424
	4M	***	0.000055
	4L	***	<0.000001
Figure 1G	3L	ns	0.926916
	4E	***	0.00021
	4M	*	0.037729
	4L	*	0.010576
Figure 1H（GP）	P+dsPxGP vs. P+dsGFP	**	0.0014
Figure 1H（HK）	P+dsPxGP vs. P+dsGFP	*	0.0151
Figure 1I	P+dsGFP vs. P+dsPxGP	***	<0.0001
	P+dsGFP vs. P+dsPxHK	***	0.0004

Figure 2

		Significant	Pvalue
Figure 2B	P+dsGFP vs. P	ns	0.9645
	P+dsGFP vs. P+dsPxGP	*	0.0216
Figure 2C	P+dsGFP vs. P	*	0.0136
	P+dsGFP vs. P+dsPxGP	ns	0.971
Figure 2D	P+dsGFP vs. P	ns	0.4131
	P+dsGFP vs. P+dsPxGP	*	0.016
Figure 2E (males)	P+dsGFP vs. P	ns	0.4293
	P+dsGFP vs. P+dsPxGP	*	0.0406
Figure 2E (females)	dsGFP vs. dsPxGP	ns	0.1396
	dsGFP vs. dsPxGP	ns	0.8518
	dsGFP vs. BC	ns	0.724781
Figure 2F (Virgin)	dsGFP vs. dsPxGP	ns	0.507702
	dsPxGP vs. BC	ns	0.153150
Figure 2F (Mated)	dsGFP♀♂ vs. dsGP♀♂	*	0.0438
	dsGFP ♀♂vs. dsGP♀ & dsGFP♂	ns	>0.9999
	dsGFP♀♂vs.dsGP♂&dsGFP♀	ns	0.8615
	dsGFP♀♂ vs. BC♀♂	ns	>0.9999
	dsGP ♀♂vs. dsGP♀ & dsGFP♂	*	0.0137
Figure 2G (Males)	P+dsGFP vs. P	ns	0.8452
	P+dsGFP vs. P+dsPxGP	***	<0.0001
Figure 2G (Famales)	P+dsGFP vs. P	ns	0.1076
	P+dsGFP vs. P+dsPxGP	ns	0.3473

Figure 3			
		Significant	P value
Figure 3D	NP: PBS vs. Bovine insulin	**	0.002781
	P: PBS vs. Bovine insulin	*	0.026145
Figure 3E	NP: PBS vs. Bovine insulin	**	0.006043
	P: PBS vs. Bovine insulin	ns	0.504827
Figure 3F	NP: PBS vs. Bovine insulin	**	0.002645
	P: PBS vs. Bovine insulin	ns	0.371862
Figure 3G	PxAKH I: NP vs. P	*	0.020342
	PxAKH II: NP vs. P	ns	0.49447
Figure 3H	P+dsGFP vs. P+dsPxAKHR	***	0.0006
Figure 3I	P+dsGFP vs. P+dsPxAKHR	ns	0.8578
Figure 3J	P+dsGFP vs. P+dsPxAKHR	ns	0.7921

Figure 4			
		Significant	P value
Figure 4A	3L: PBS vs. Venom	ns	0.1317
	3L: PBS vs. CvBVf	***	<0.0001
	4M: PBS vs. Teratocytes	**	0.0027
Figure 4B	PxGP: PBS vs. Venom	ns	0.6206
	PxGP: PBS vs. CvBVf	ns	0.7681
	PxHK: PBS vs. Venom	ns	0.7582
	PxHK: PBS vs. CvBVf	***	<0.0001
	PxTPS: PBS vs. Venom	ns	0.8981
	PxTPS: PBS vs. CvBVf	ns	0.5634
Figure 4C	PxGP: PBS vs. Teratocytes	*	0.016043
	PxHK: PBS vs. Teratocytes	ns	0.617489
	PxTPS: PBS vs. Teratocytes	ns	0.880097
Figure 4F	PBS vs. 0.005	***	0.0006
	PBS vs. 0.05	**	0.0047
	PBS vs. 0.5	***	0.001
Figure 4G	PxGP: PBS vs. sCvsNPF	*	0.01665
	PxHK: PBS vs. sCvsNPF	ns	0.814383
	PxTPS: PBS vs. sCvsNPF	ns	0.500714
Figure 4I	PBS vs. sCvsNPF	*	0.0377

Figure 5			
		Significant	P value
Figure 5A	NP vs. P: 3L	**	0.007326
	NP vs. P: 4E	***	0.000198
	NP vs. P: 4M	*	0.012679
	NP vs. P: 4L	***	0.000332
Figure 5B	PBS vs. 0.005 sPxsNPF-1	ns	>0.9999
	PBS vs. 0.05 sPxsNPF-1	ns	0.9978
	PBS vs. 0.5 sPxsNPF-1	**	0.0013
	PBS vs. 0.5 sPxsNPF-2	ns	0.7454
	PBS vs. 0.5 sPxsNPF-3	ns	0.9987
Figure 5D	NP vs. P	***	<0.0001
Figure 5F	NP vs. P	***	<0.0001
Figure 5G	PBS vs. sPxsNPF-1: PxGP	ns	0.967927
	PBS vs. sPxsNPF-1: PxHK	***	0.000094
	PBS vs. sPxsNPF-1: PxTPS	ns	0.96048
Figure 5H	P+dsGFP vs. P+dsPxsNPF	**	0.0024
Figure 5I	P+dsGFP vs. P+dsPxsNPF	***	<0.0001
Figure 5J	P+dsGFP vs. P+dsPxsNPF: PxGP	ns	0.846925
	P+dsGFP vs. P+dsPxsNPF: PxHK	**	0.008272
	P+dsGFP vs. P+dsPxsNPF: PxTPS	ns	0.858836
Figure 5K	3M: PBS vs. Venom	ns	0.9016
	3M: PBS vs. CvBVf	*	0.0389
	4E: PBS vs. Teratocytes	ns	>0.9999
Figure 5L	NP vs. P	***	<0.000001
	NP vs. sPxsNPF-1	***	<0.000001
	NP vs. sCvsNPF	ns	0.963205

Figure 6			
		Significant	P value
Figure 6A	dsGFP vs. dsPxsNPFR	**	0.0046
Figure 6B	dsGFP vs. dsPxsNPFR	ns	0.9613
	dsGFP vs. dsGFP+sPxsNPF-1	*	0.0146
	dsGFP vs. dsPxsNPFR+sPxsNPF-1	ns	0.9999
	dsPxsNPFR vs. dsPxsNPFR+sPxsNPF-1	ns	0.8747
	dsGFP+sPxsNPF-1 vs. dsPxsNPFR+sPxsNPF-1	**	0.0079
Figure 6C	dsGFP vs. dsPxsNPFR	ns	0.842
	dsGFP vs. dsGFP+sCvsNPF	*	0.0317
	dsGFP vs. dsPxsNPFR+sCvsNPF	ns	>0.9999
	dsPxsNPFR vs. dsPxsNPFR+sCvsNPF	ns	0.6844
	dsGFP+sCvsNPF vs. dsPxsNPFR+sCvsNPF	*	0.0344
Figure 6G	sPxsNPF-1 vs. CK	***	<0.0001
	sPxsNPF-1 vs. sPxsNPF-1+PTX	ns	0.1606
	sPxsNPF-1 vs. sPxsNPF-1+U73	***	<0.0001
Figure 6H	sCvsNPF vs. CK	****	<0.0001
	sCvsNPF vs. sCvsNPF+PTX	****	<0.0001
	sCvsNPF vs. sCvsNPF+U73	ns	0.8253
