# Supplementary figures and images for: Dual interference with host neuropeptide signaling allows parasitoid wasp to hijack host sugar metabolism
Source: EMBO J. 2025 Nov 26;45(6):2030–50. doi: 10.1038/s44318-025-00636-5 (PMC12992715; doi:10.1038/s44318-025-00636-5)

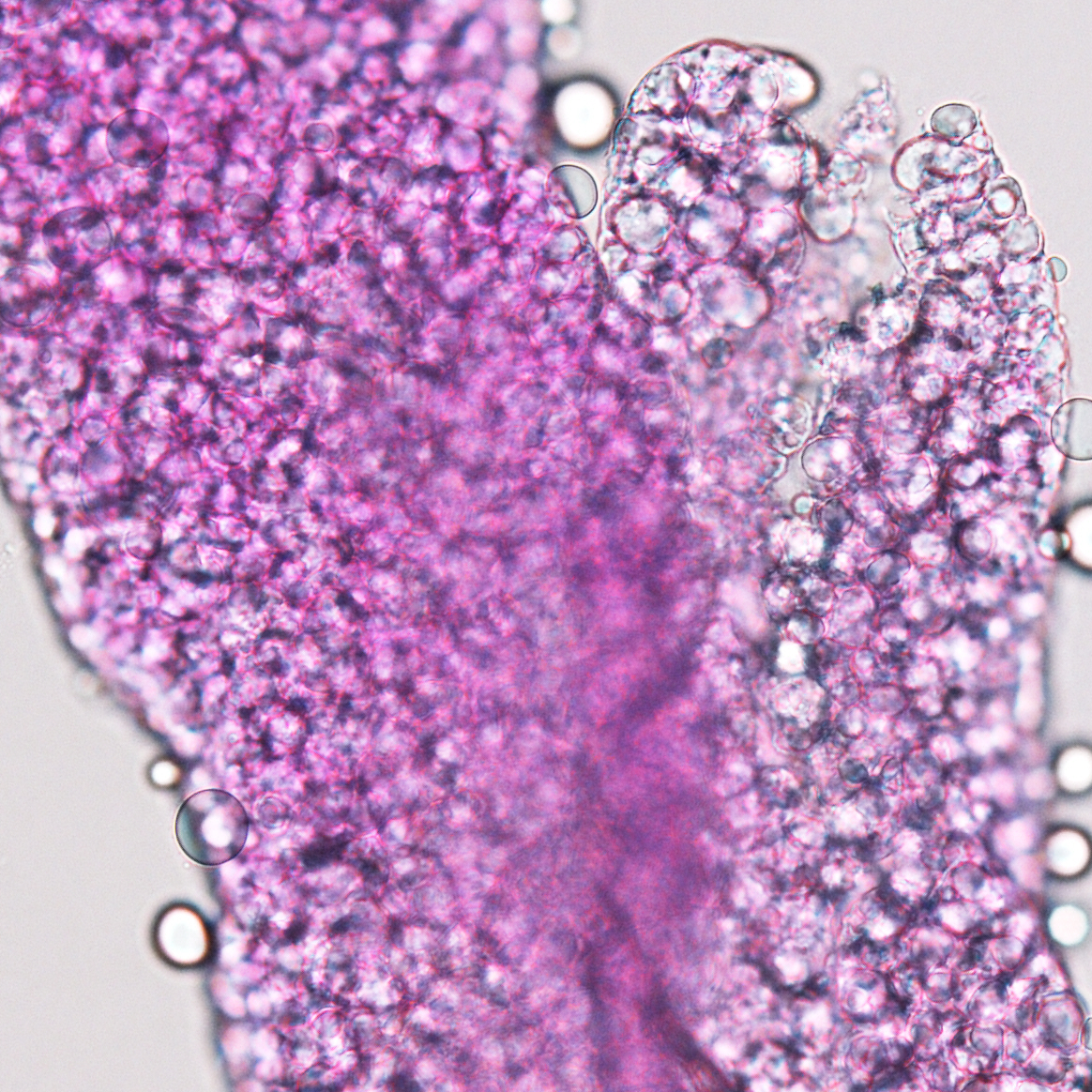

Supplement: Supplementary file 5 — Source data Fig. 1 [file 44318_2025_636_MOESM5_ESM.zip › Figure 1/Figure 1F/Figure 1F_P_4E.tif]

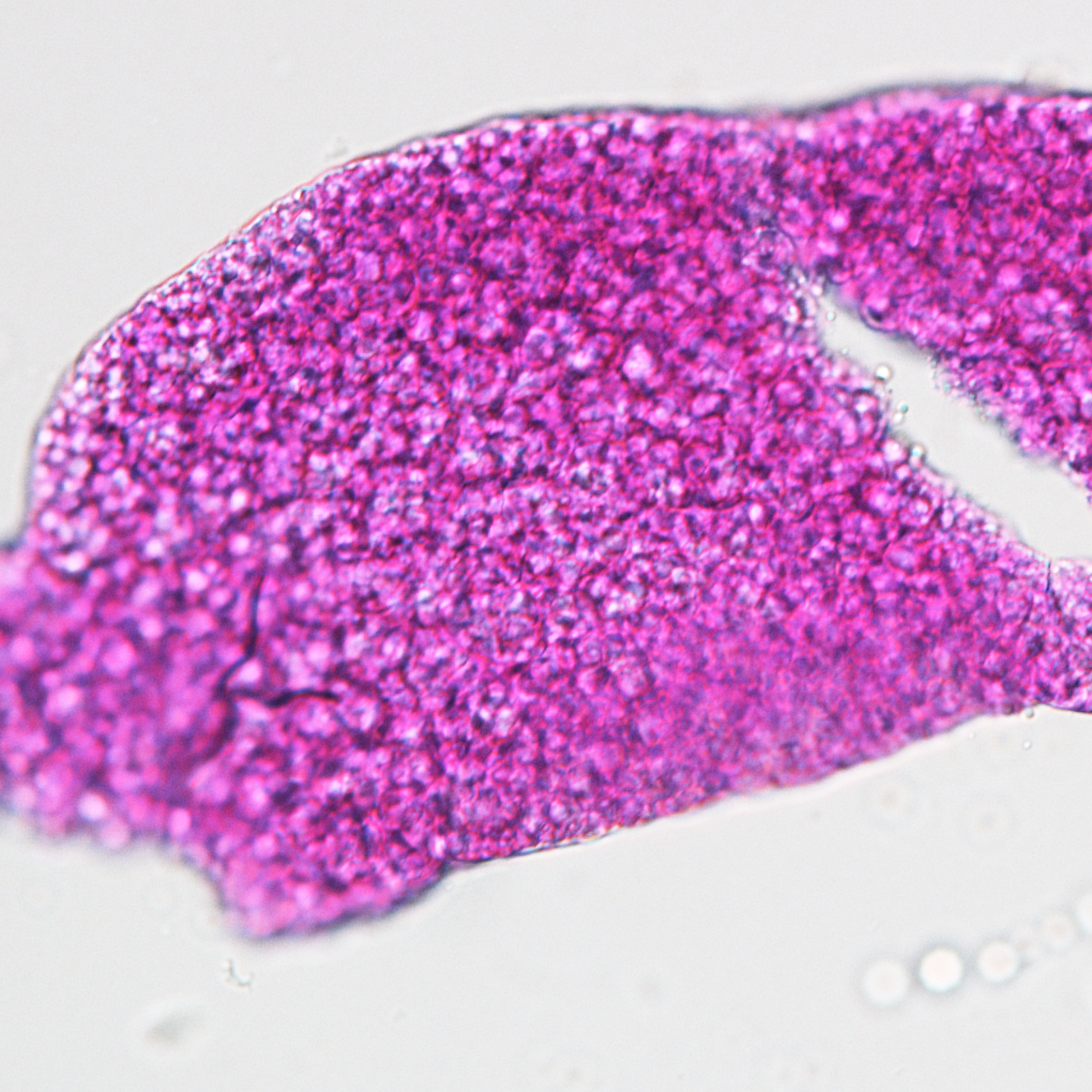

Supplement: Supplementary file 5 — Source data Fig. 1 [file 44318_2025_636_MOESM5_ESM.zip › Figure 1/Figure 1F/Figure 1F_NP_4E.tif]

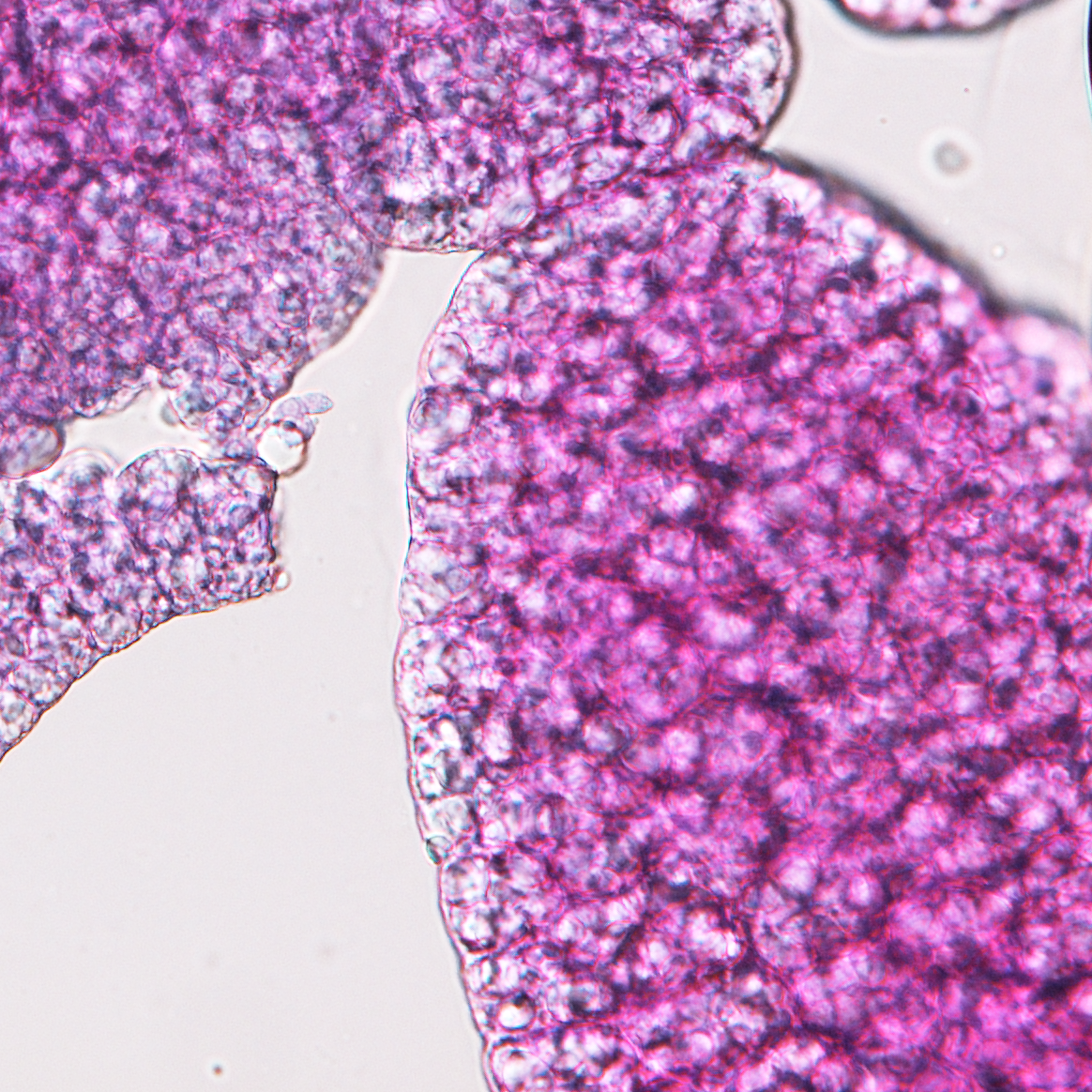

Supplement: Supplementary file 5 — Source data Fig. 1 [file 44318_2025_636_MOESM5_ESM.zip › Figure 1/Figure 1F/Figure 1F_NP_4M.tif]

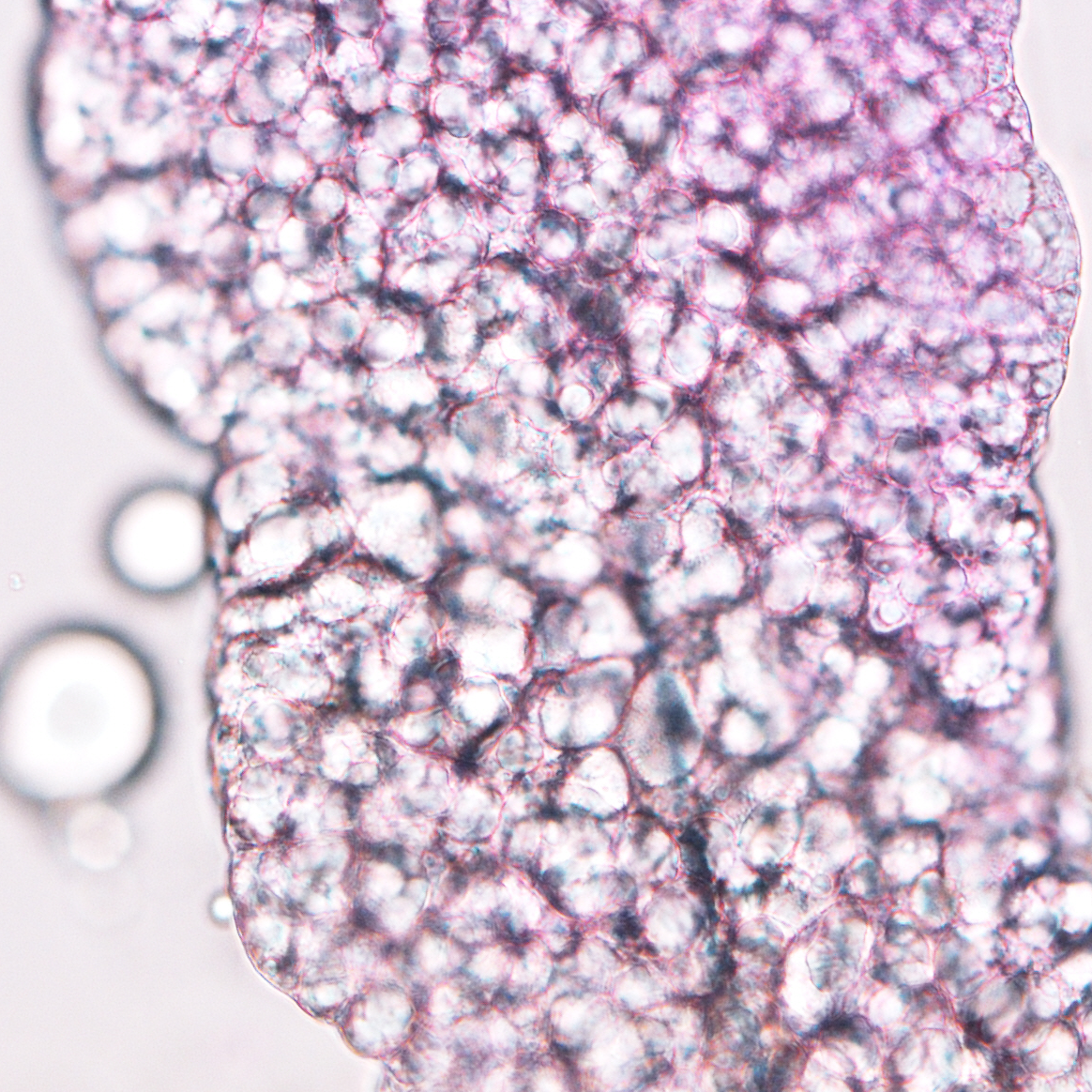

Supplement: Supplementary file 5 — Source data Fig. 1 [file 44318_2025_636_MOESM5_ESM.zip › Figure 1/Figure 1F/Figure 1F_P_4M.tif]

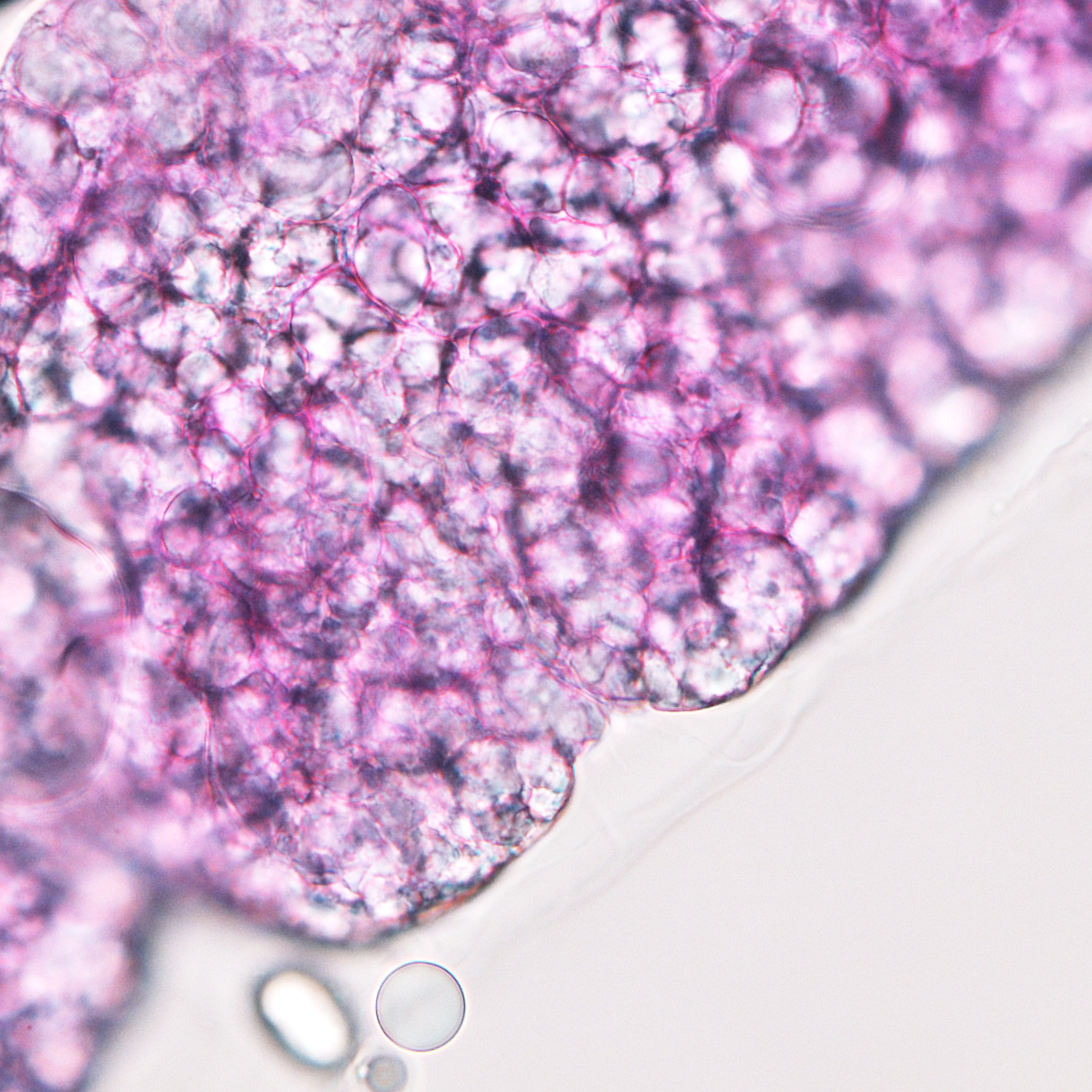

Supplement: Supplementary file 5 — Source data Fig. 1 [file 44318_2025_636_MOESM5_ESM.zip › Figure 1/Figure 1F/Figure 1F_P_4L.tif]

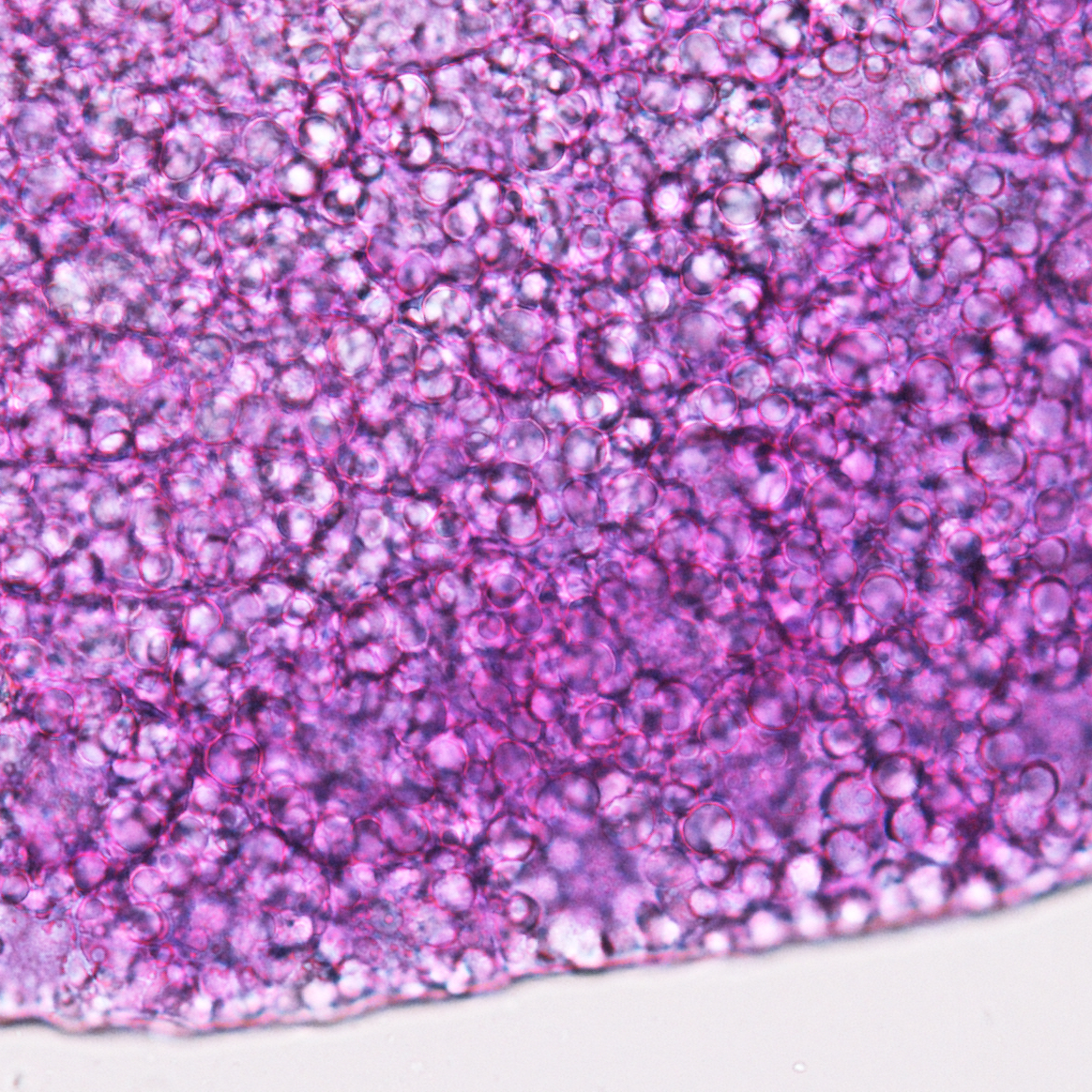

Supplement: Supplementary file 5 — Source data Fig. 1 [file 44318_2025_636_MOESM5_ESM.zip › Figure 1/Figure 1F/Figure 1F_NP_4L.tif]

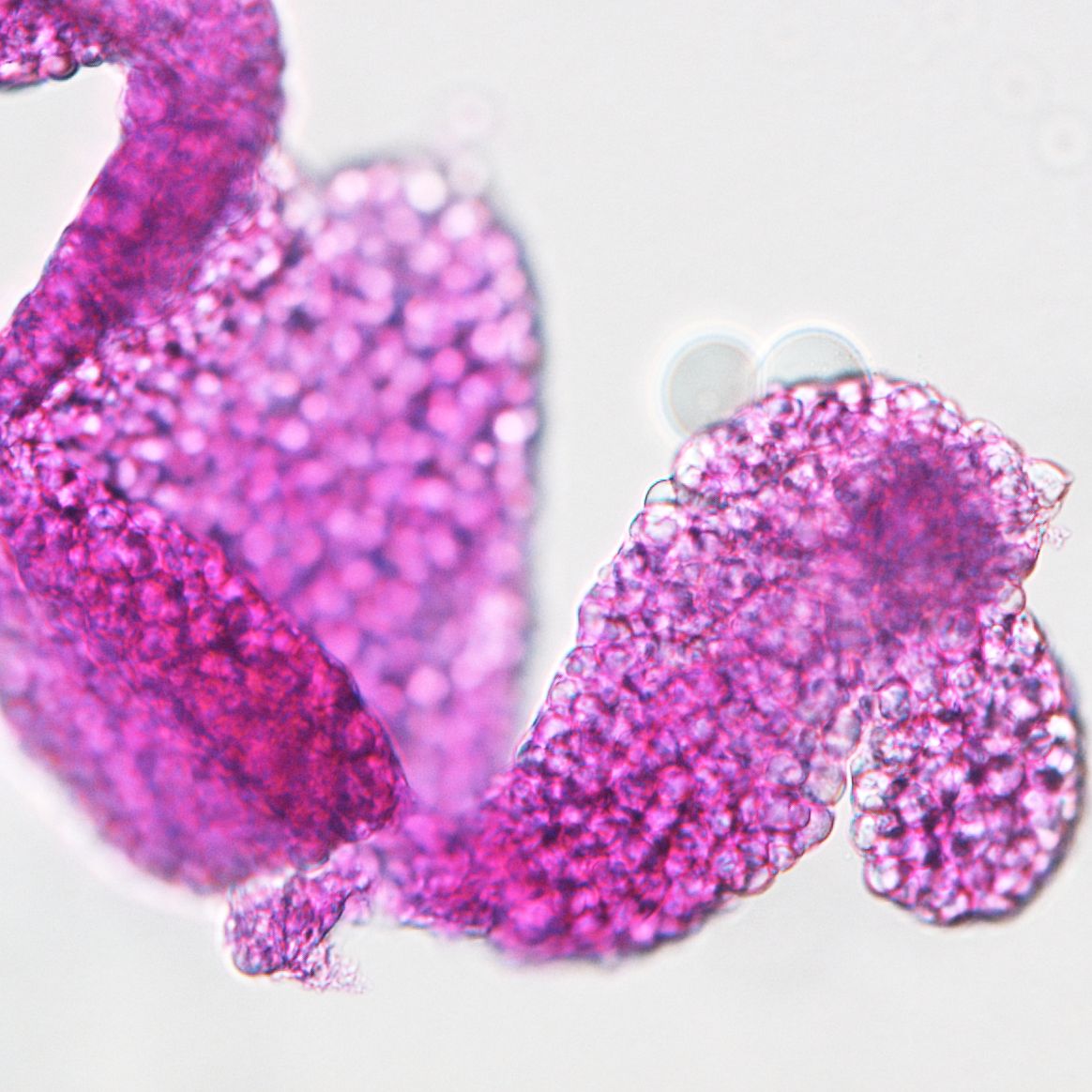

Supplement: Supplementary file 5 — Source data Fig. 1 [file 44318_2025_636_MOESM5_ESM.zip › Figure 1/Figure 1F/Figure 1F_NP_3L.tif]

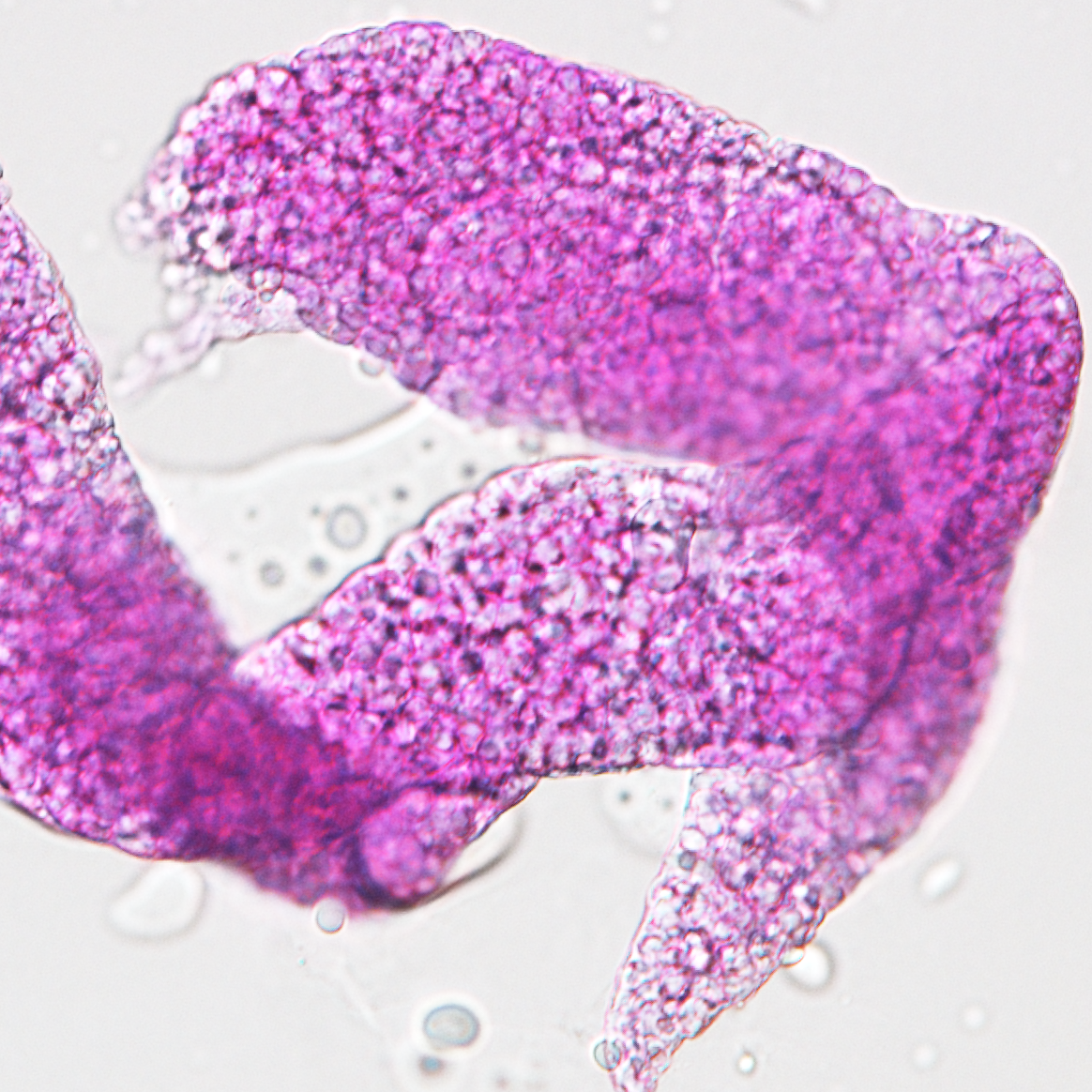

Supplement: Supplementary file 5 — Source data Fig. 1 [file 44318_2025_636_MOESM5_ESM.zip › Figure 1/Figure 1F/Figure 1F_P_3L.tif]

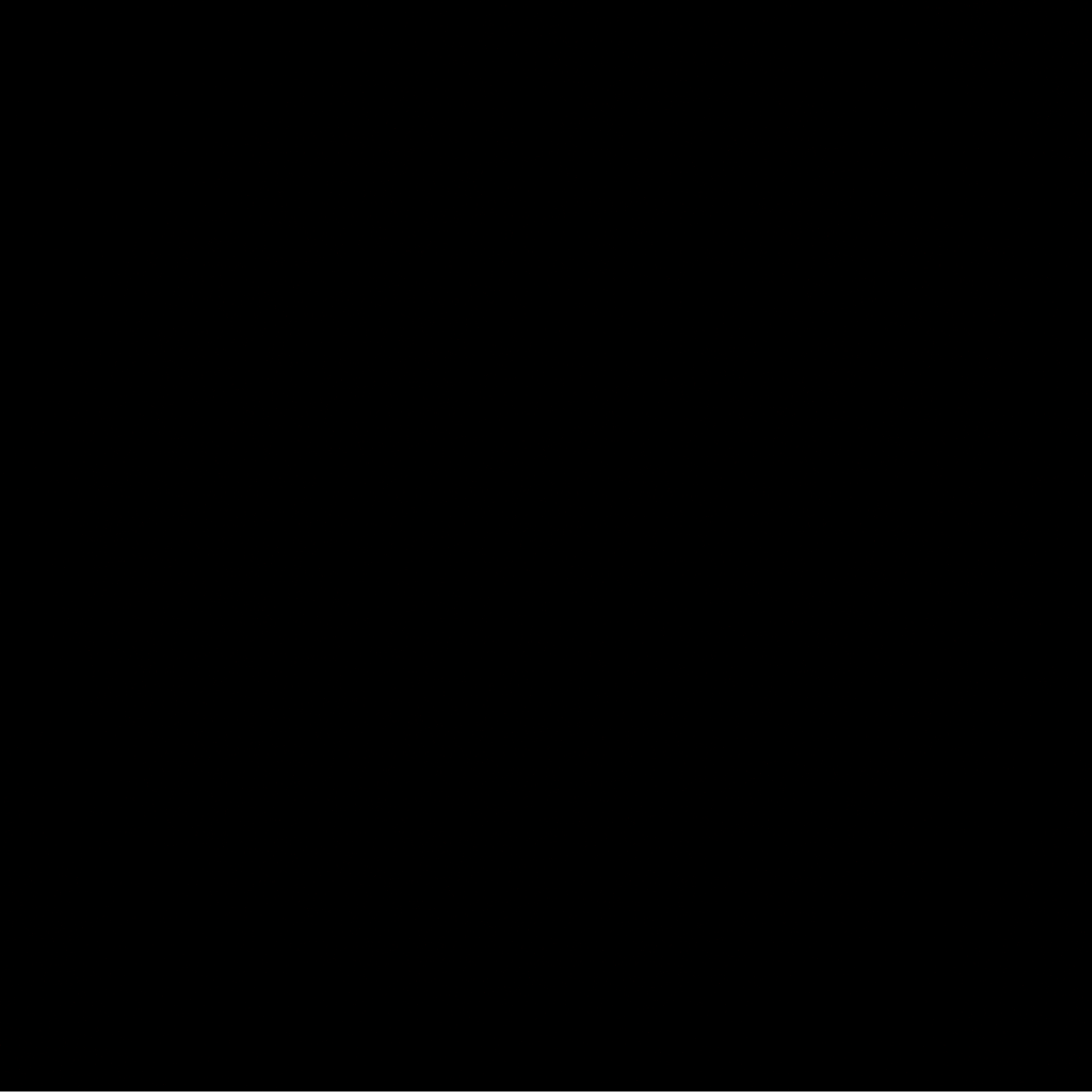

Supplement: Supplementary file 8 — Source data Fig. 4 [file 44318_2025_636_MOESM8_ESM.zip › Figure 4/Figure 4D/Figure 4D-negative control-CvsNPF.tif]

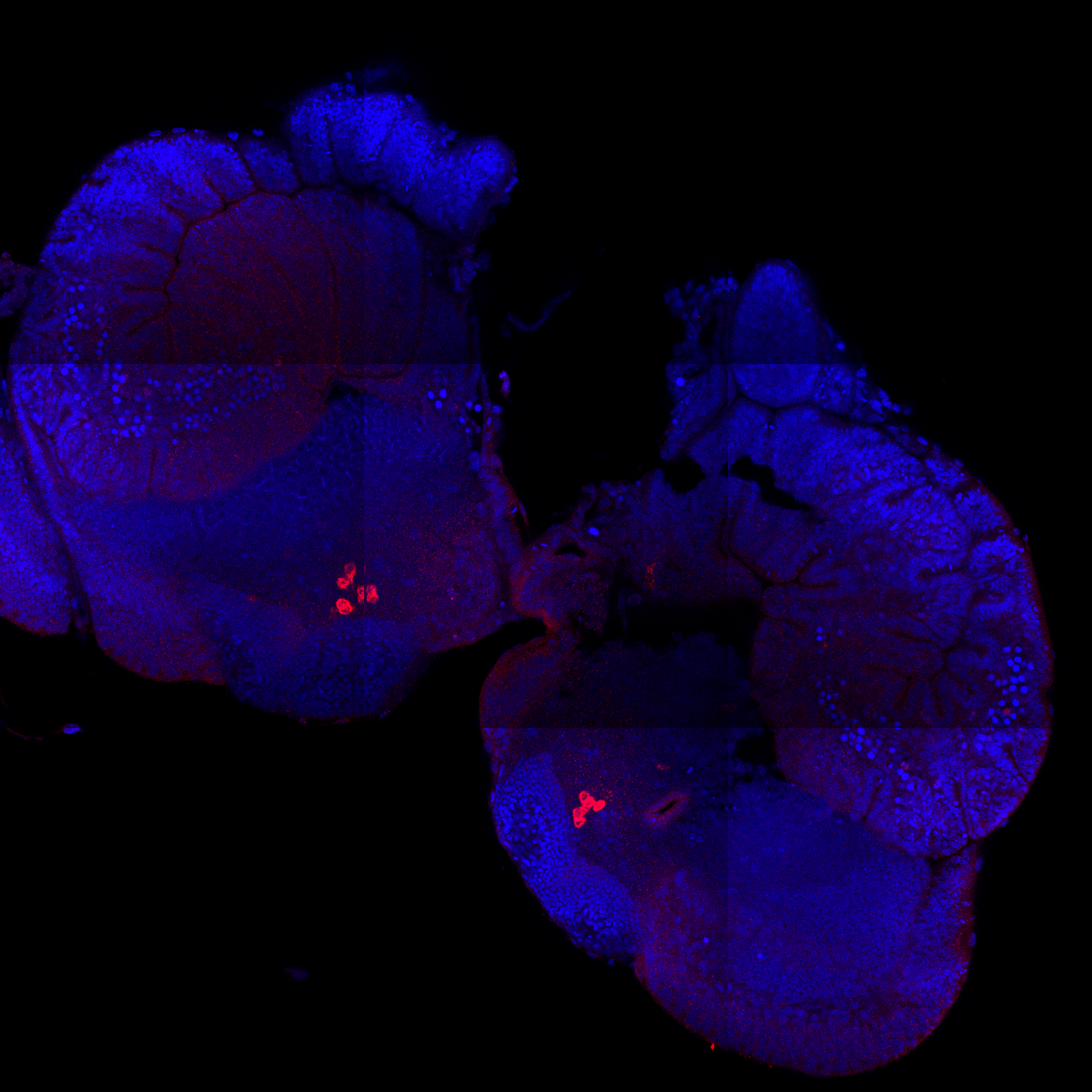

Supplement: Supplementary file 8 — Source data Fig. 4 [file 44318_2025_636_MOESM8_ESM.zip › Figure 4/Figure 4D/Figure 4D-positive control-merged.tif]

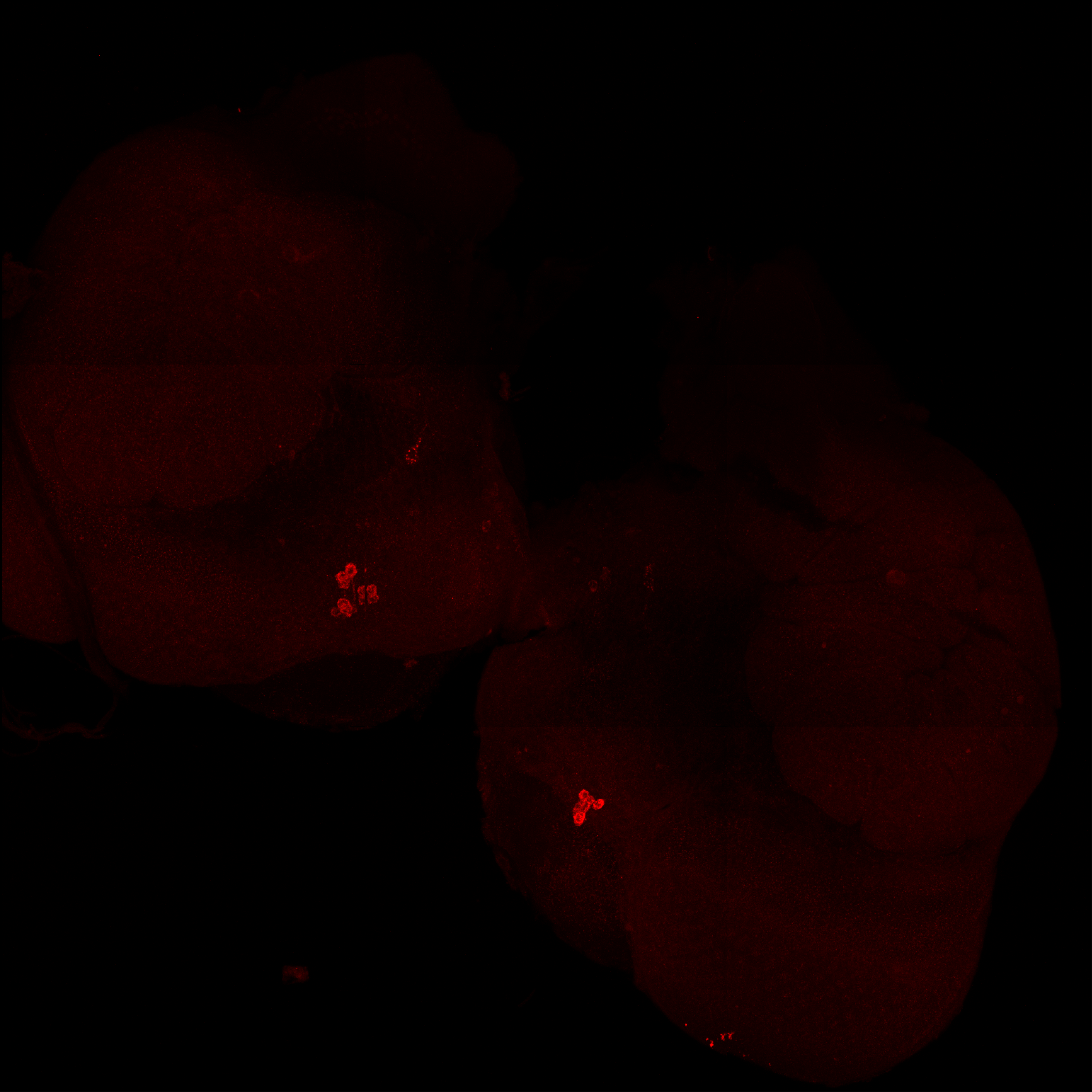

Supplement: Supplementary file 8 — Source data Fig. 4 [file 44318_2025_636_MOESM8_ESM.zip › Figure 4/Figure 4D/Figure 4D-positive control_CvsNPF.tif]

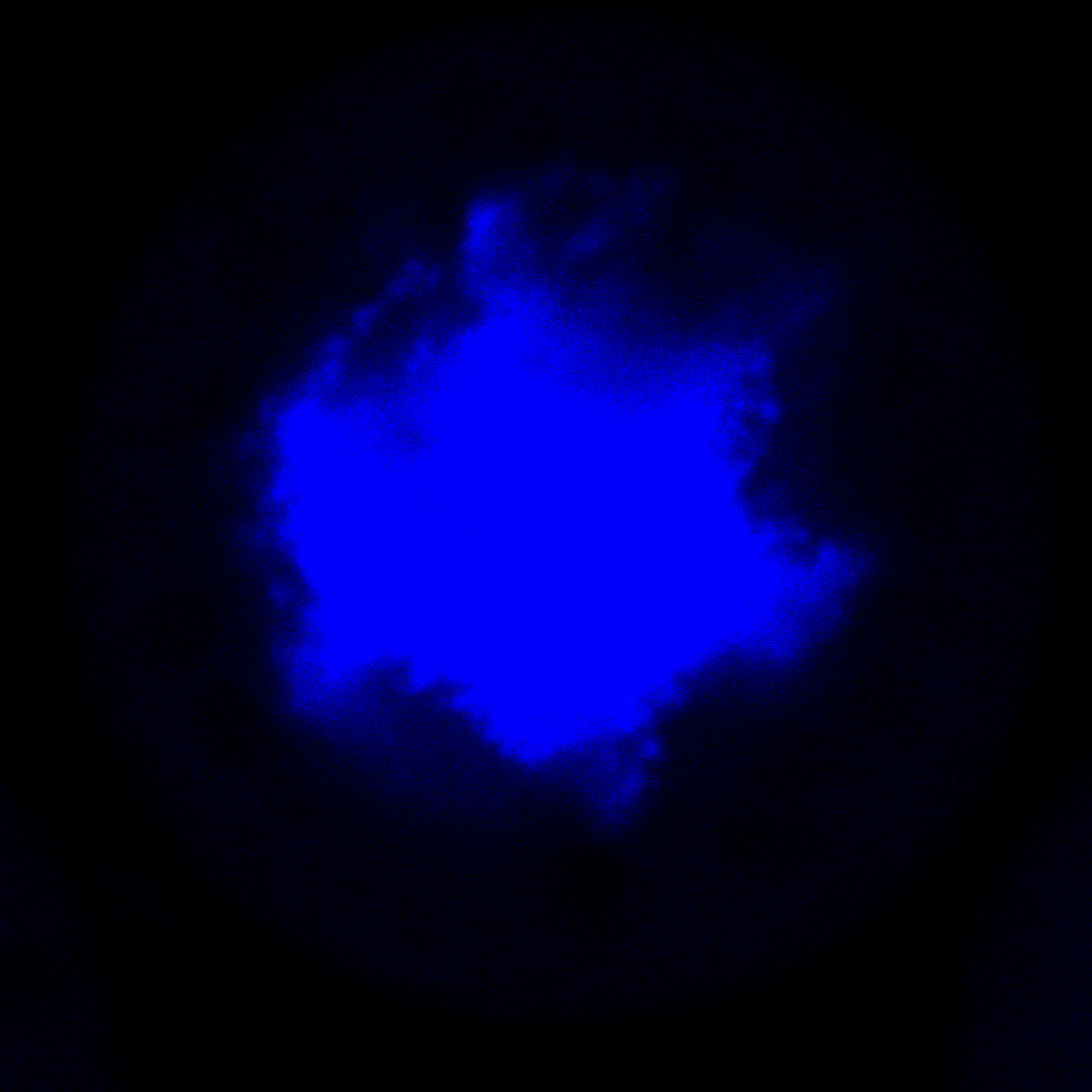

Supplement: Supplementary file 8 — Source data Fig. 4 [file 44318_2025_636_MOESM8_ESM.zip › Figure 4/Figure 4D/Figure 4D-negative control-Merged.tif]

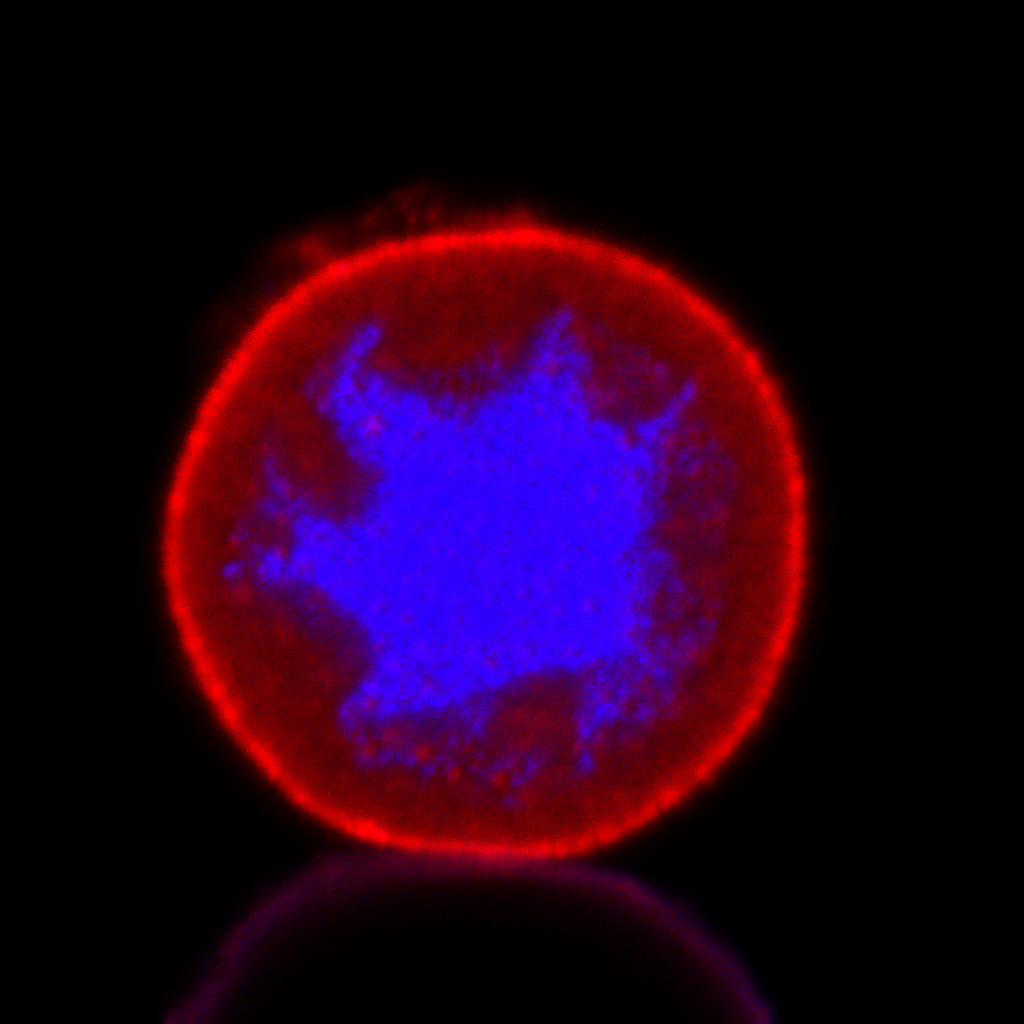

Supplement: Supplementary file 8 — Source data Fig. 4 [file 44318_2025_636_MOESM8_ESM.zip › Figure 4/Figure 4D/Figure 4D-teratocytes-merged.tif]

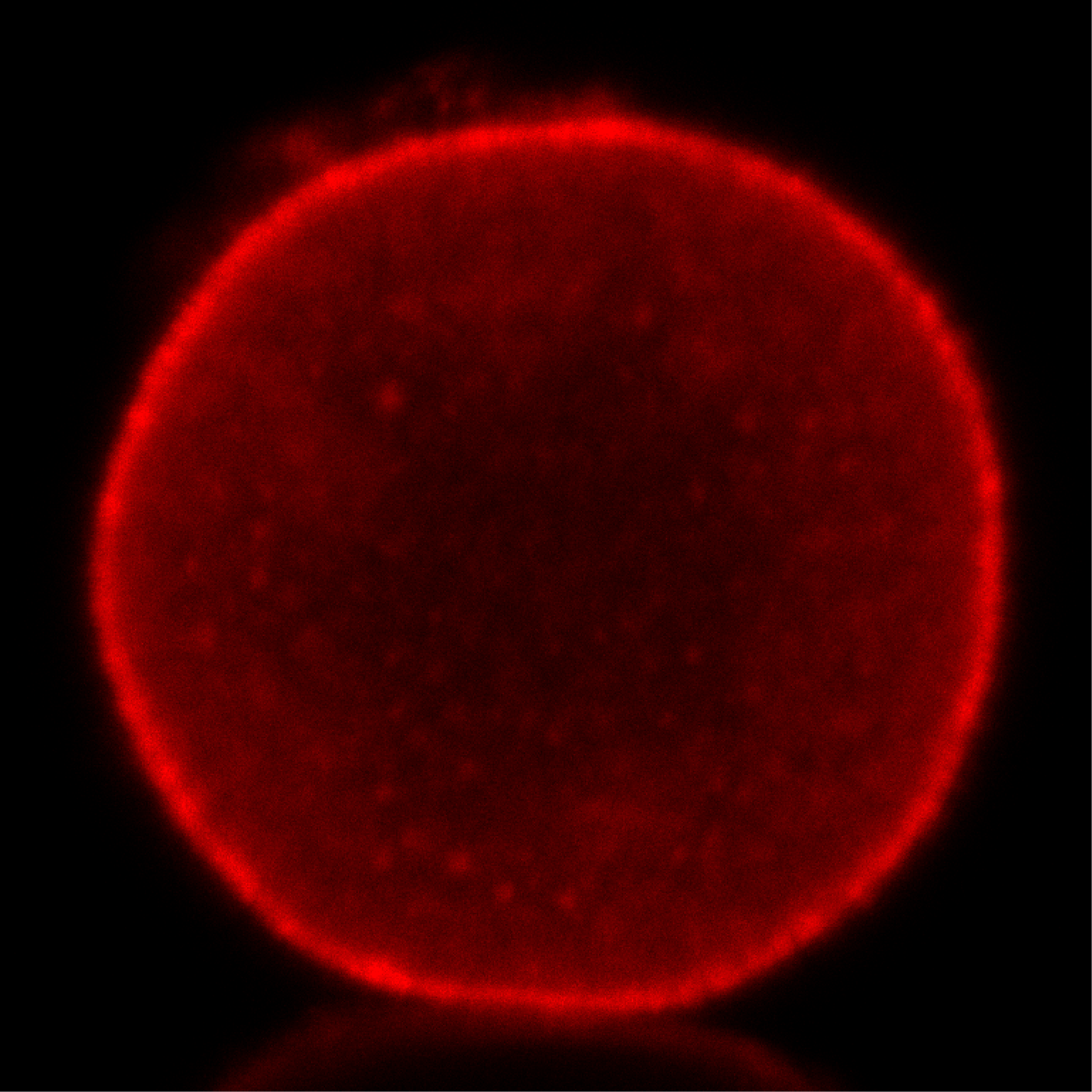

Supplement: Supplementary file 8 — Source data Fig. 4 [file 44318_2025_636_MOESM8_ESM.zip › Figure 4/Figure 4D/Figure 4D-teratocytes-CvsNPF.tif]

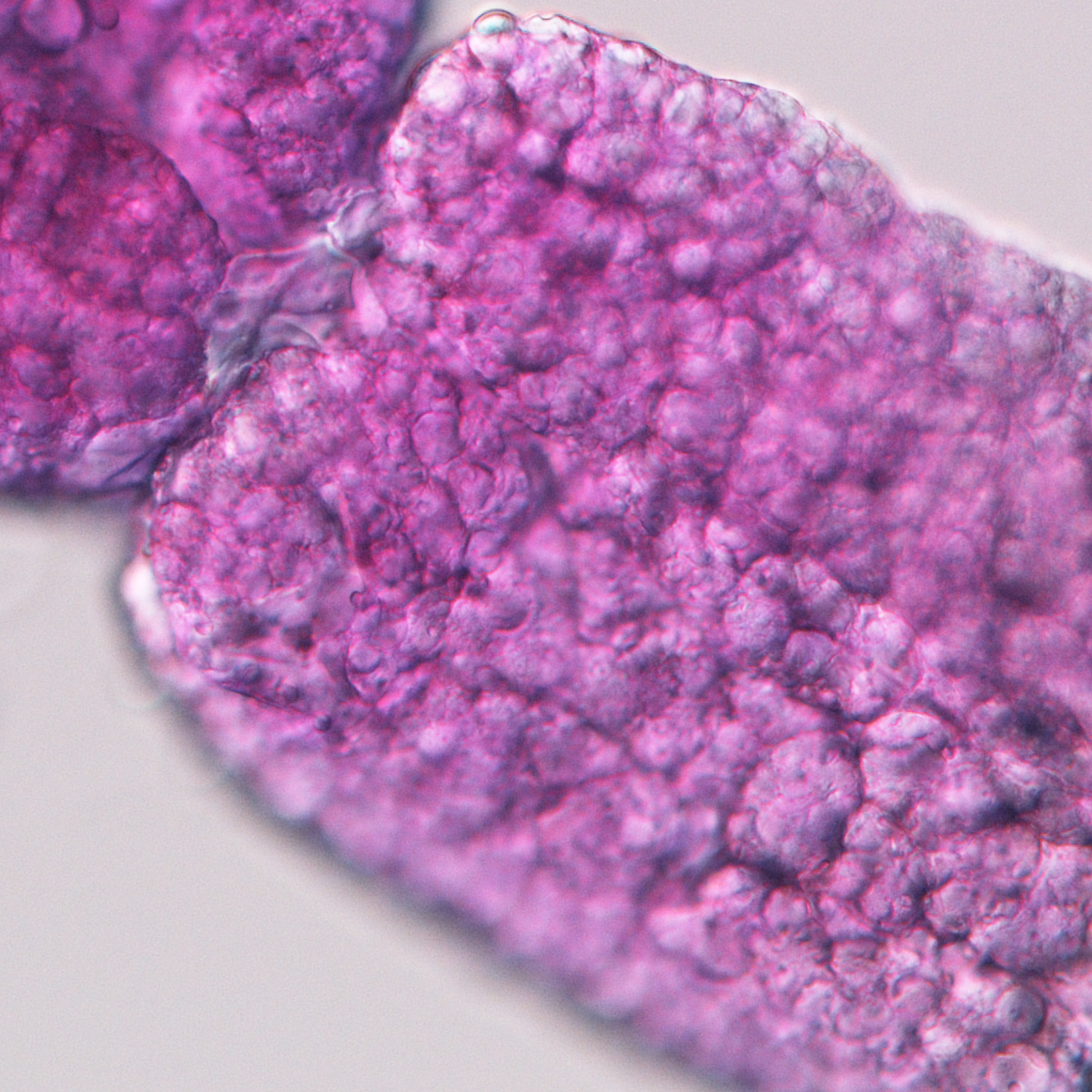

Supplement: Supplementary file 8 — Source data Fig. 4 [file 44318_2025_636_MOESM8_ESM.zip › Figure 4/Figure 4H/Figure 4H_sCvsNPF.tif]

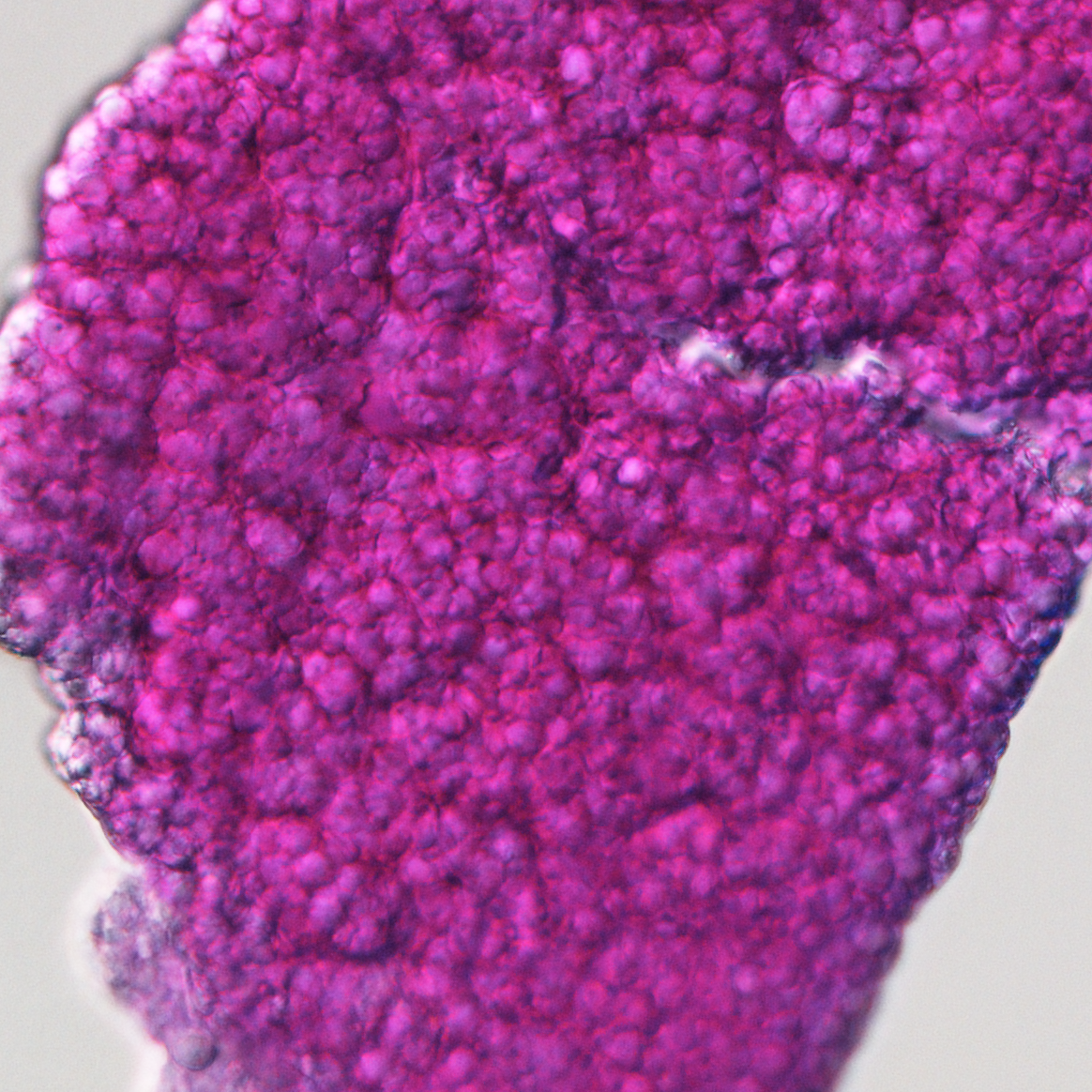

Supplement: Supplementary file 8 — Source data Fig. 4 [file 44318_2025_636_MOESM8_ESM.zip › Figure 4/Figure 4H/Figure 4H_PBS.tif]

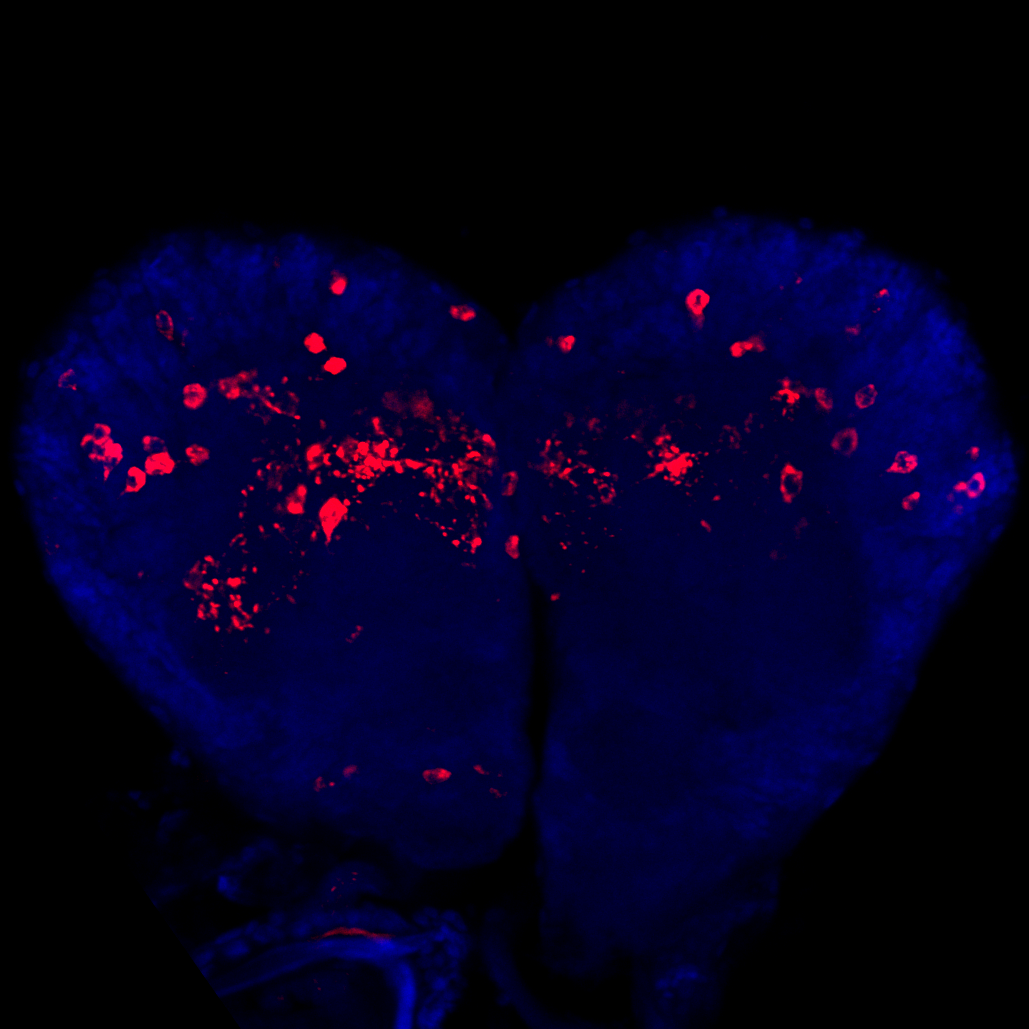

Supplement: Supplementary file 9 — Source data Fig. 5 [file 44318_2025_636_MOESM9_ESM.zip › Figure 5C/Figure 5C.tif]

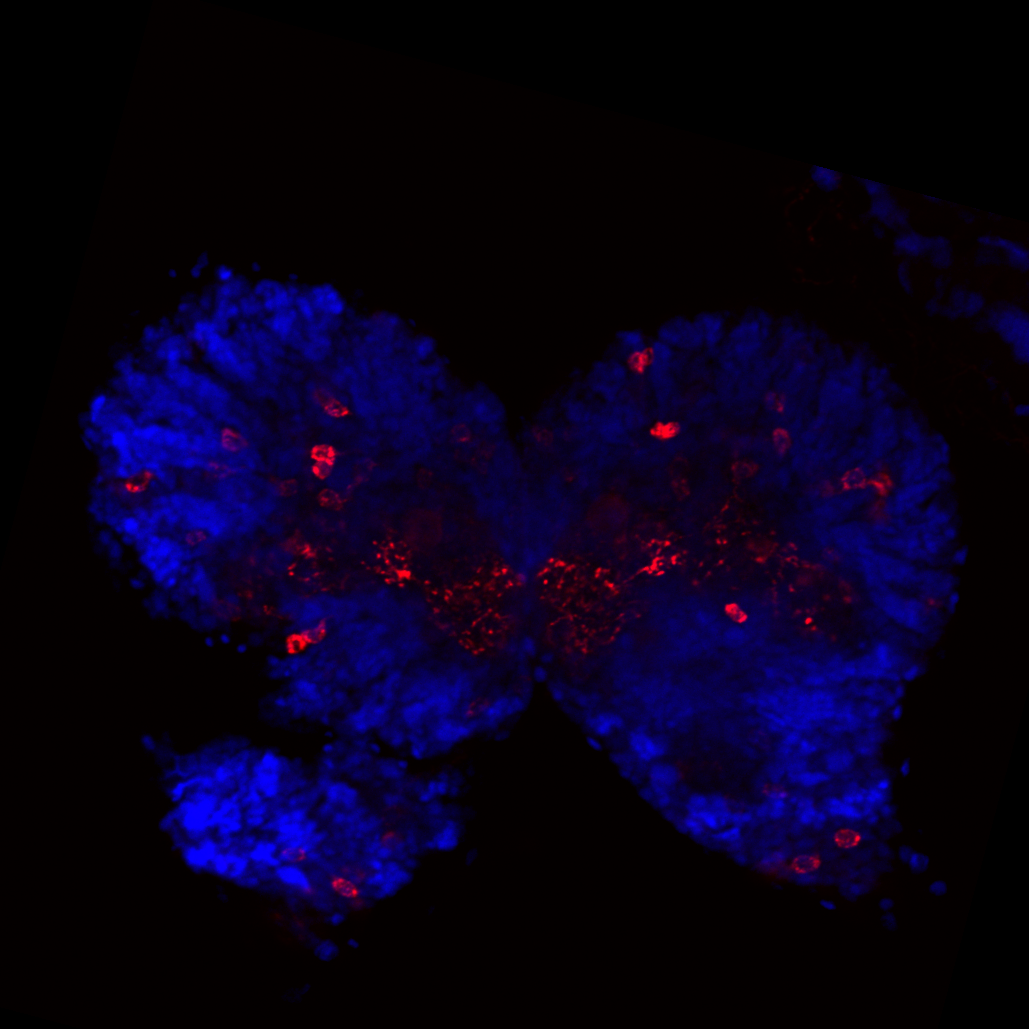

Supplement: Supplementary file 9 — Source data Fig. 5 [file 44318_2025_636_MOESM9_ESM.zip › Figure 5C/Figure 5C-NP.tif]

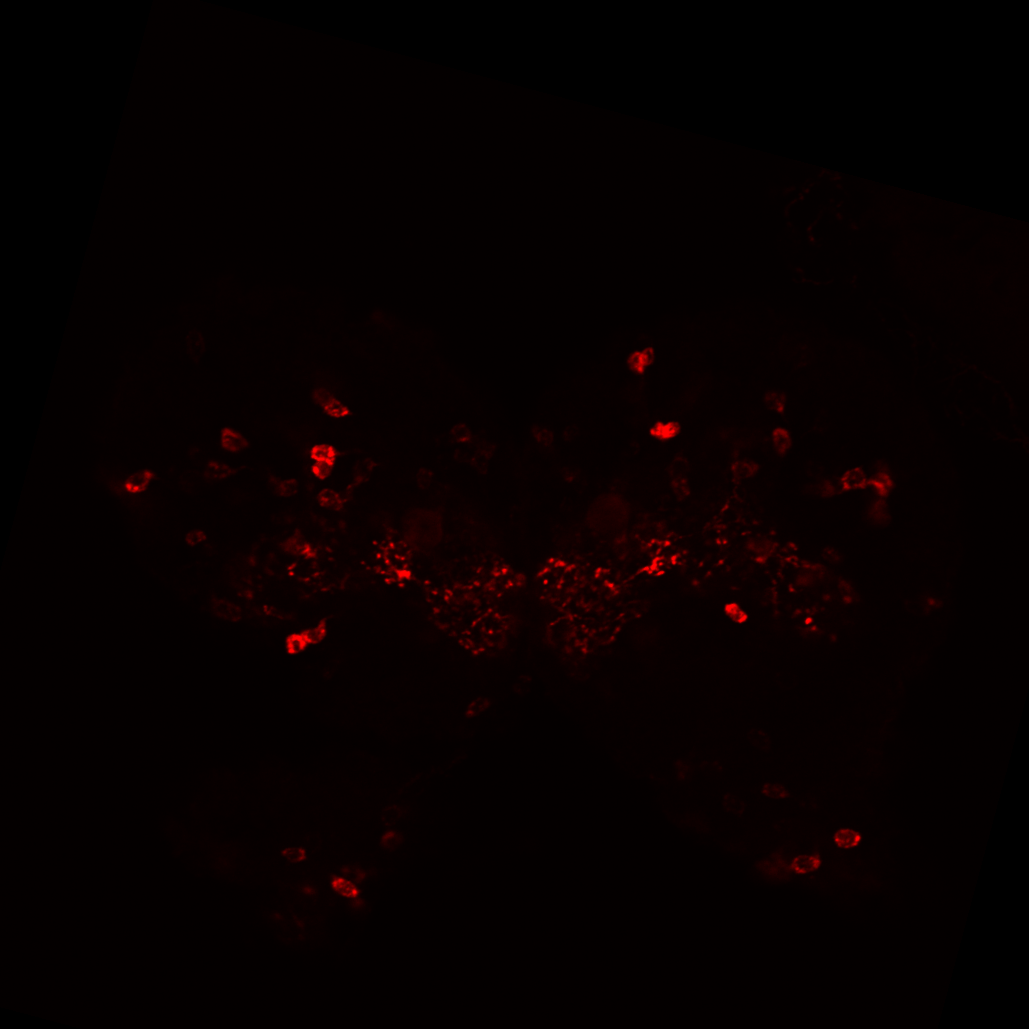

Supplement: Supplementary file 9 — Source data Fig. 5 [file 44318_2025_636_MOESM9_ESM.zip › Figure 5C/Figure 5C-NP-PxsNPF.tif]

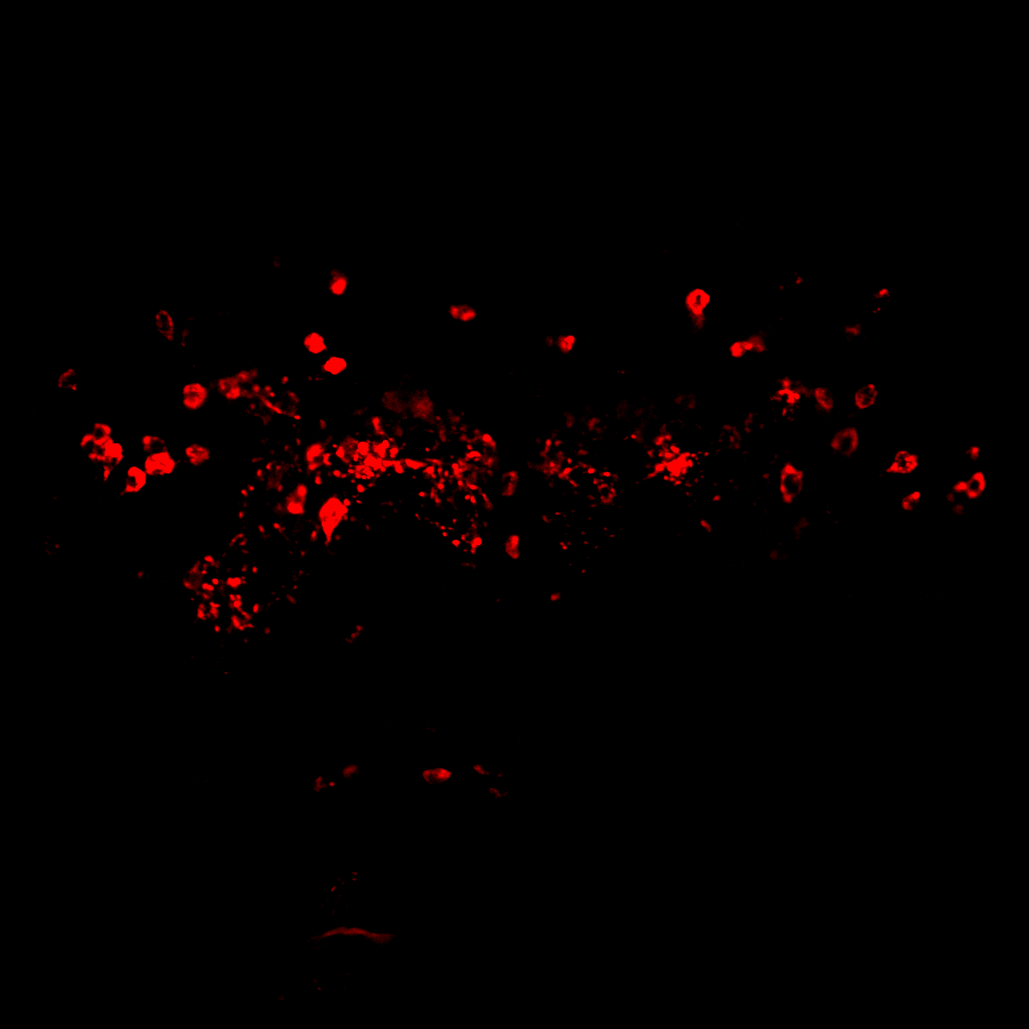

Supplement: Supplementary file 9 — Source data Fig. 5 [file 44318_2025_636_MOESM9_ESM.zip › Figure 5C/Figure 5C-P-PxsNPF.tif]

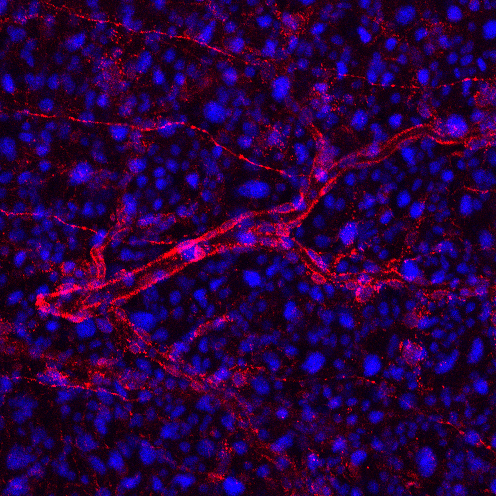

Supplement: Supplementary file 9 — Source data Fig. 5 [file 44318_2025_636_MOESM9_ESM.zip › Figure 5E/Figure 5E-NP.tif]

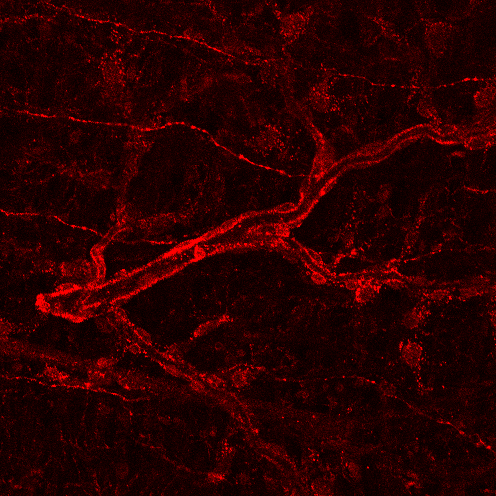

Supplement: Supplementary file 9 — Source data Fig. 5 [file 44318_2025_636_MOESM9_ESM.zip › Figure 5E/Figure 5E-NP-PxsNPF.tif]

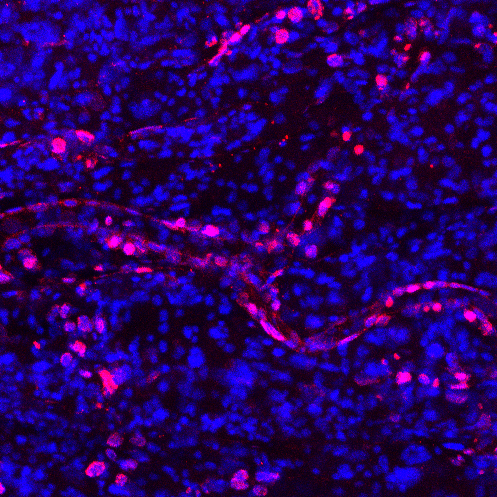

Supplement: Supplementary file 9 — Source data Fig. 5 [file 44318_2025_636_MOESM9_ESM.zip › Figure 5E/Figure 5E-P.tif]

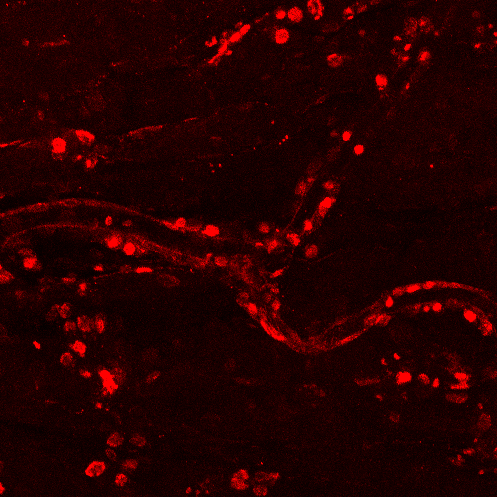

Supplement: Supplementary file 9 — Source data Fig. 5 [file 44318_2025_636_MOESM9_ESM.zip › Figure 5E/Figure 5E-P-PxsNPF.tif]

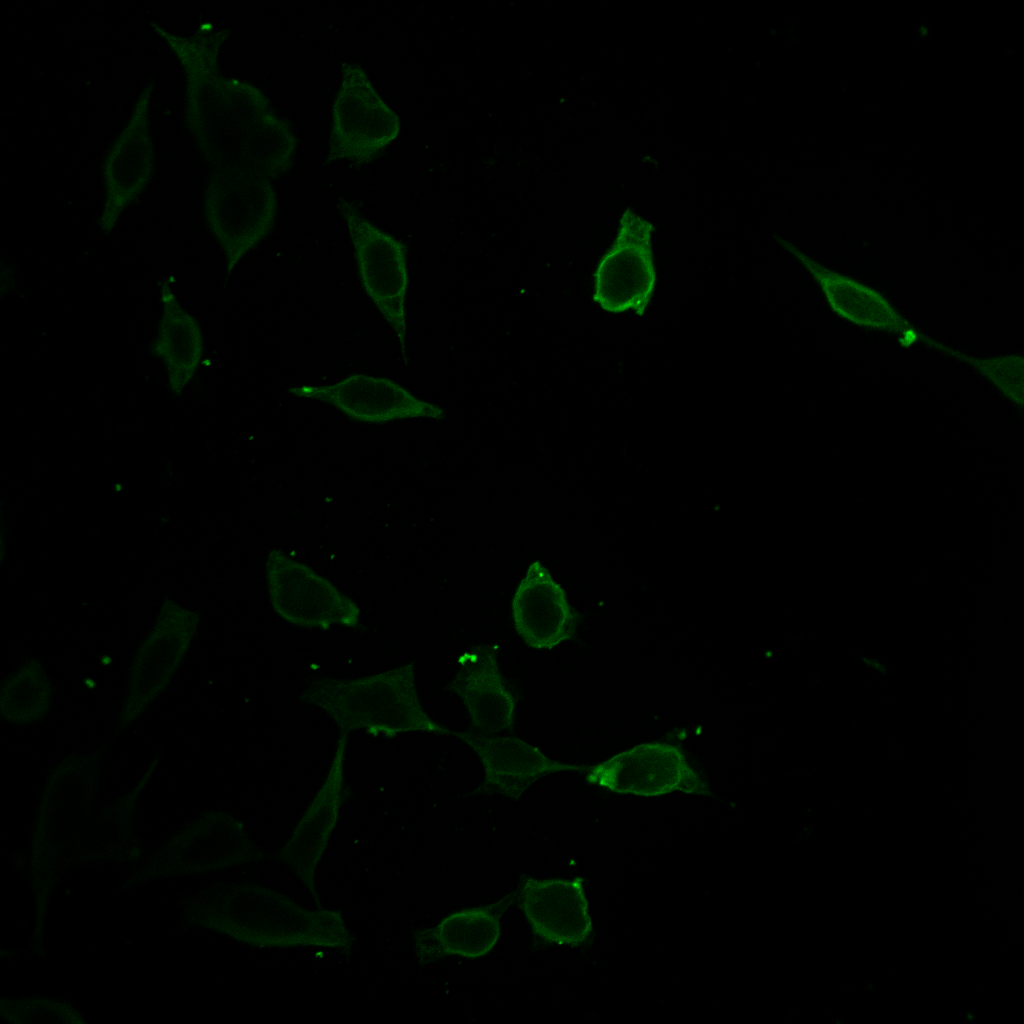

Supplement: Supplementary file 11 — Appendix Figure Source Data [file 44318_2025_636_MOESM11_ESM.zip › Figure S7/Figure S7B/Figure S7B-PxsNPFR-PxsNPFR.tif]

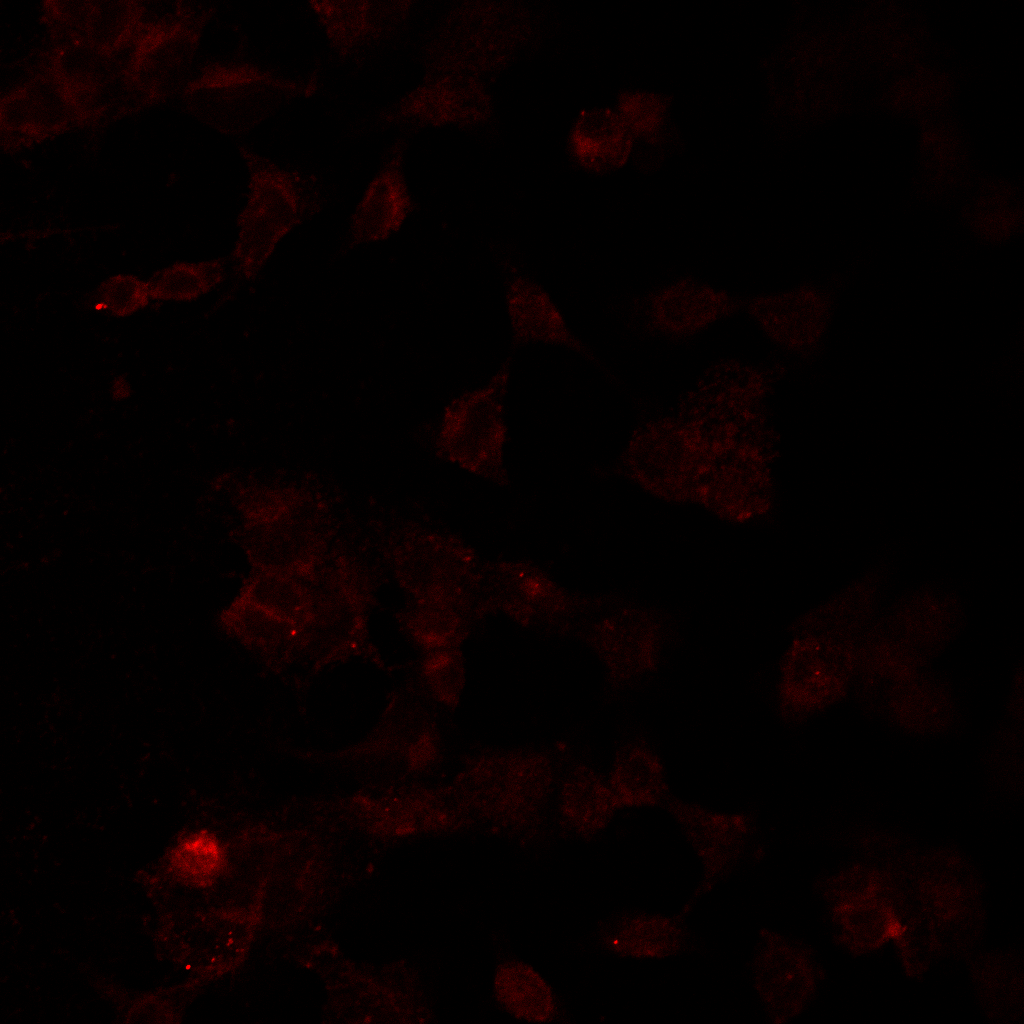

Supplement: Supplementary file 11 — Appendix Figure Source Data [file 44318_2025_636_MOESM11_ESM.zip › Figure S7/Figure S7B/Figure S7B-HEK293-Membrane.tif]

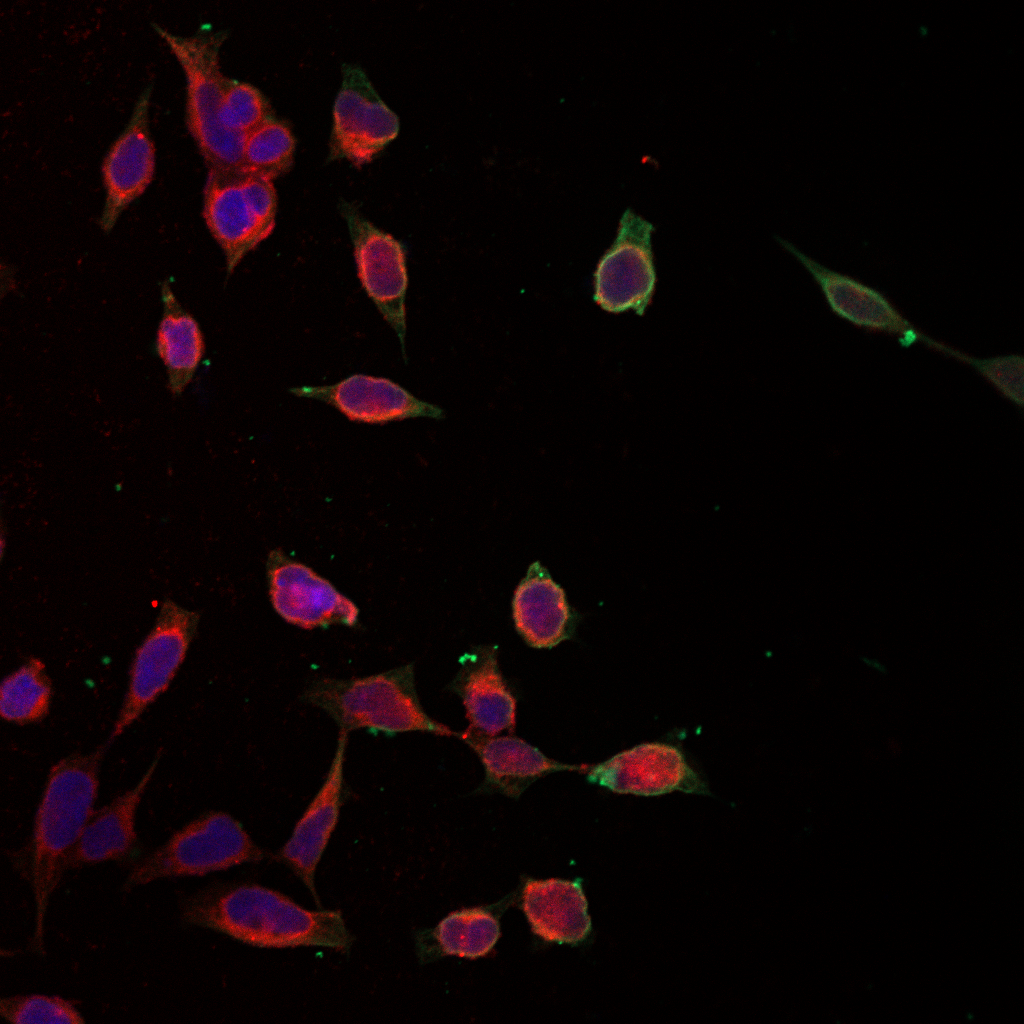

Supplement: Supplementary file 11 — Appendix Figure Source Data [file 44318_2025_636_MOESM11_ESM.zip › Figure S7/Figure S7B/Figure S7B-PxsNPFR-Merge3.tif]

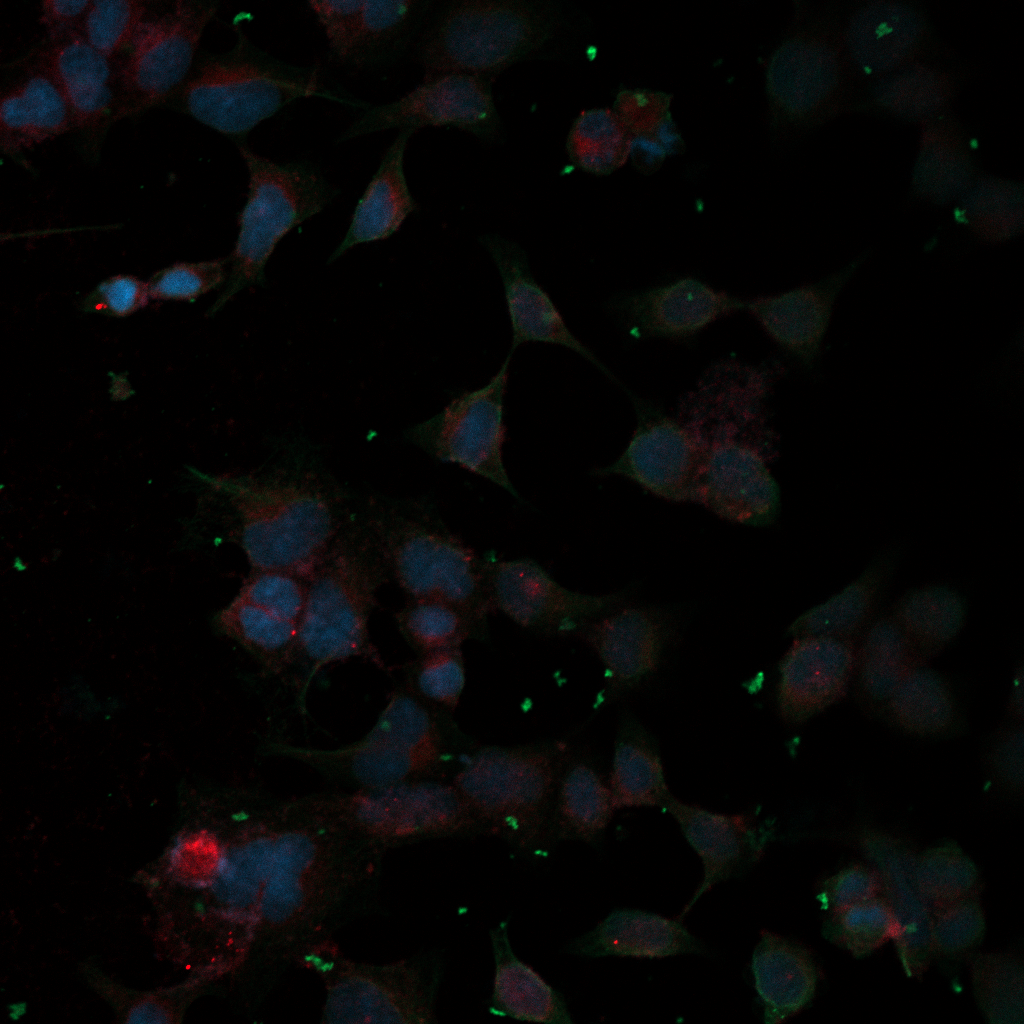

Supplement: Supplementary file 11 — Appendix Figure Source Data [file 44318_2025_636_MOESM11_ESM.zip › Figure S7/Figure S7B/Figure S7B-HEK293-Merged.tif]

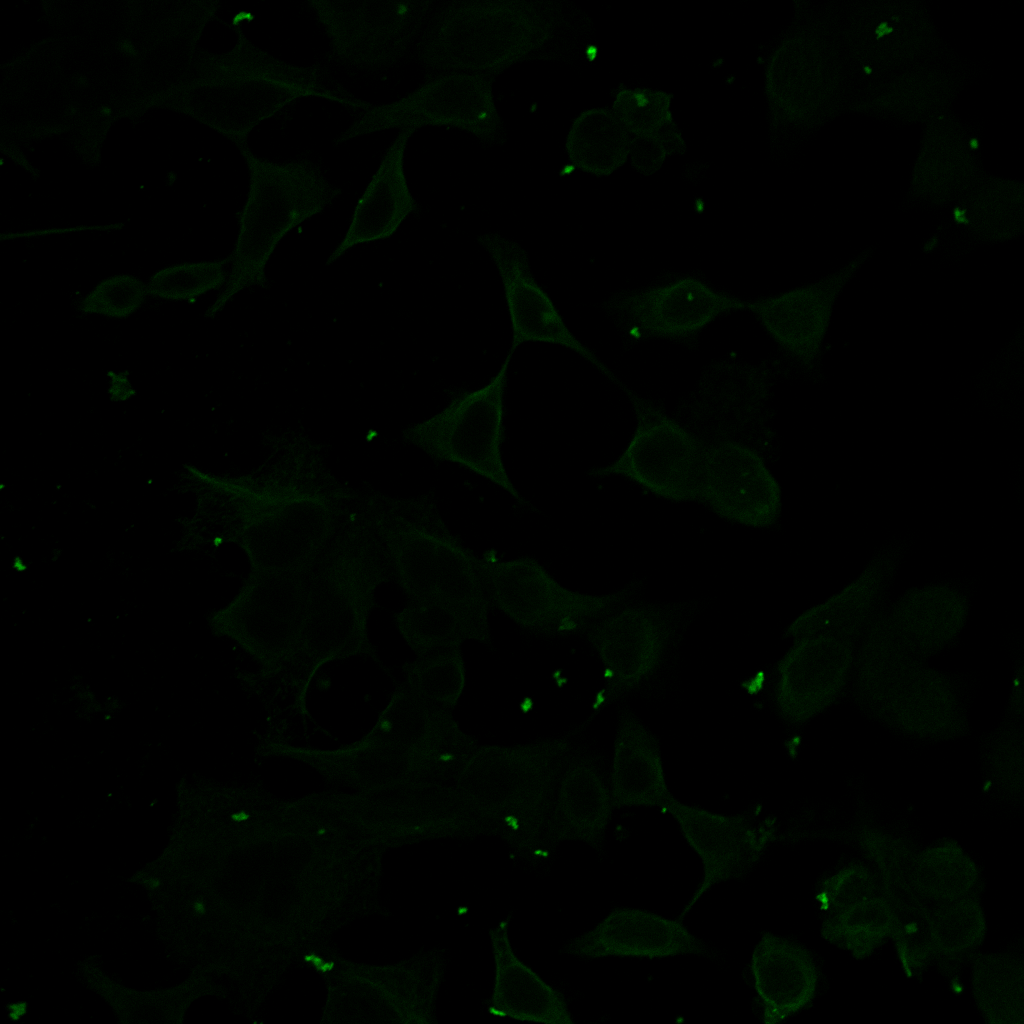

Supplement: Supplementary file 11 — Appendix Figure Source Data [file 44318_2025_636_MOESM11_ESM.zip › Figure S7/Figure S7B/Figure S7B-HEK293-PxsNPFR.tif]

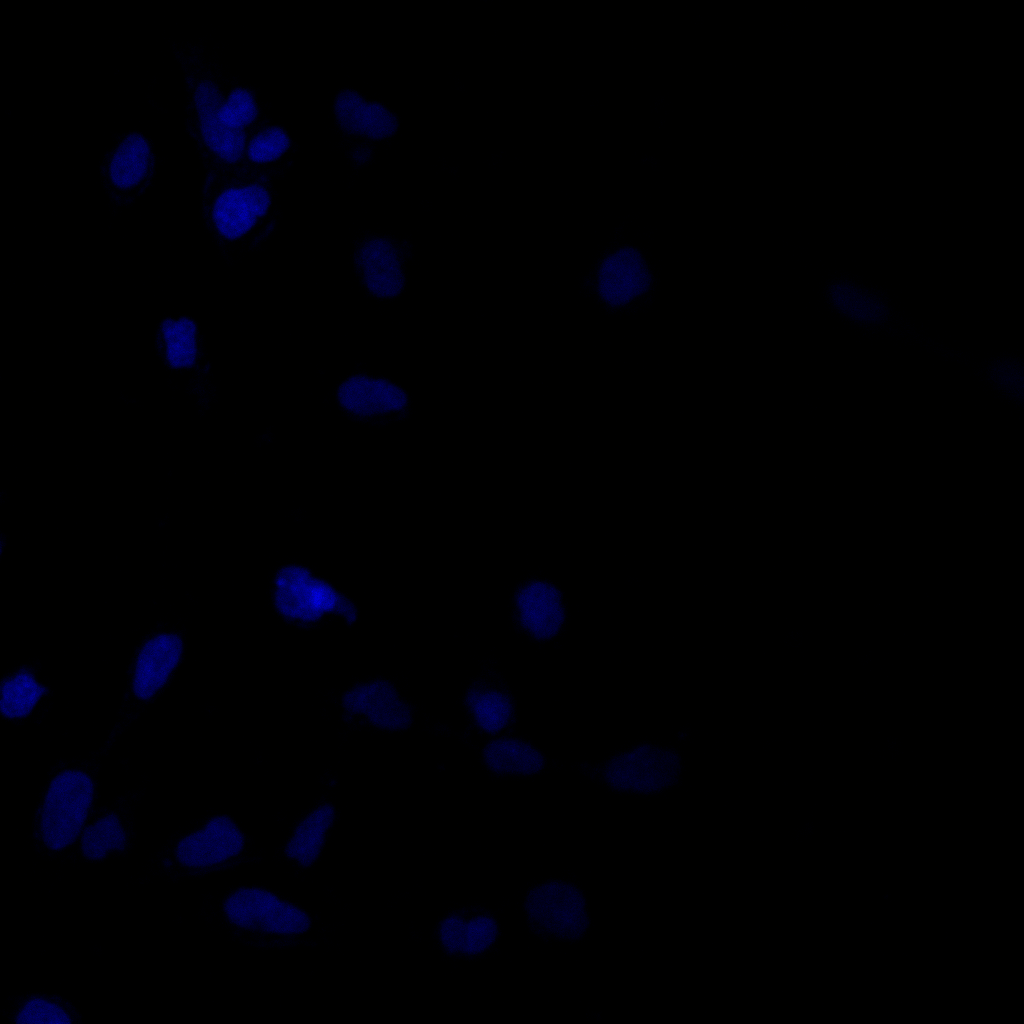

Supplement: Supplementary file 11 — Appendix Figure Source Data [file 44318_2025_636_MOESM11_ESM.zip › Figure S7/Figure S7B/Figure S7B-PxsNPFR-DAPI.tif]

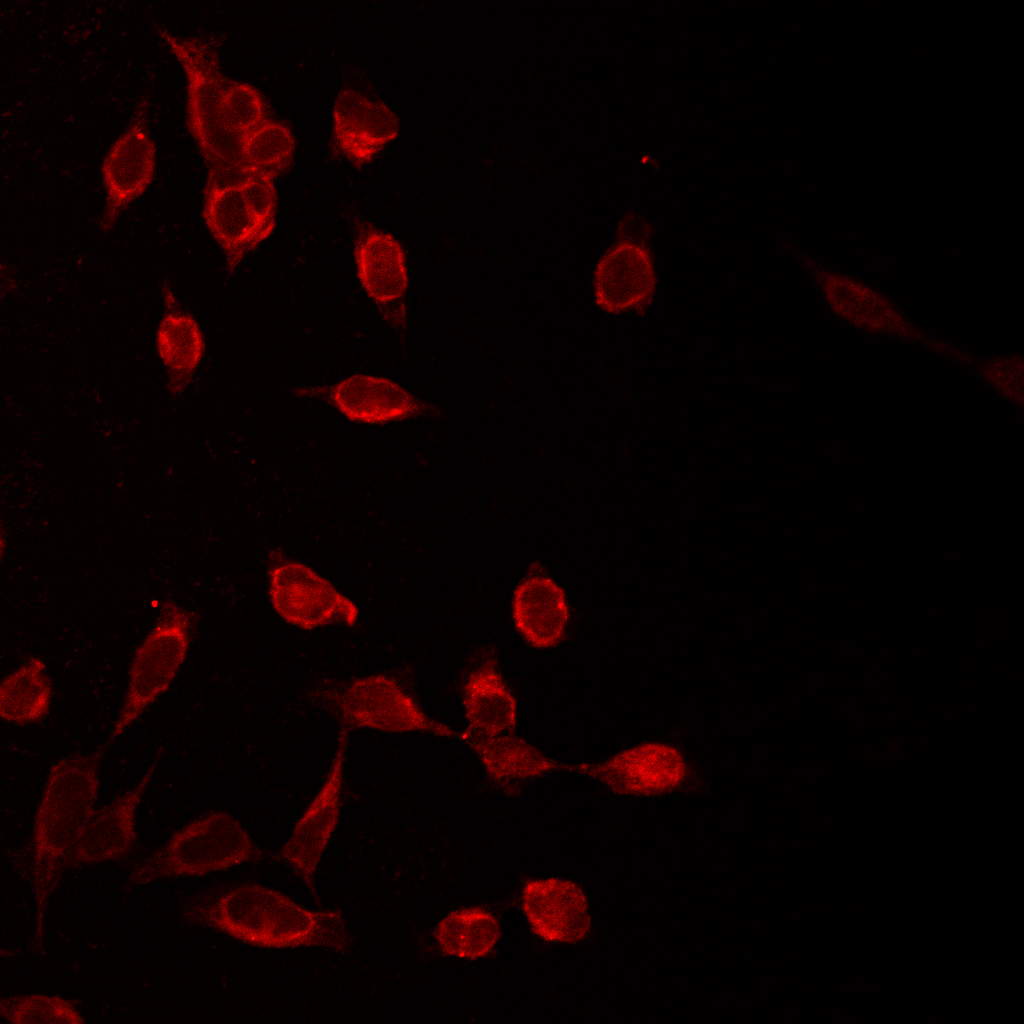

Supplement: Supplementary file 11 — Appendix Figure Source Data [file 44318_2025_636_MOESM11_ESM.zip › Figure S7/Figure S7B/Figure S7B-PxsNPFR-Membrane.tif]

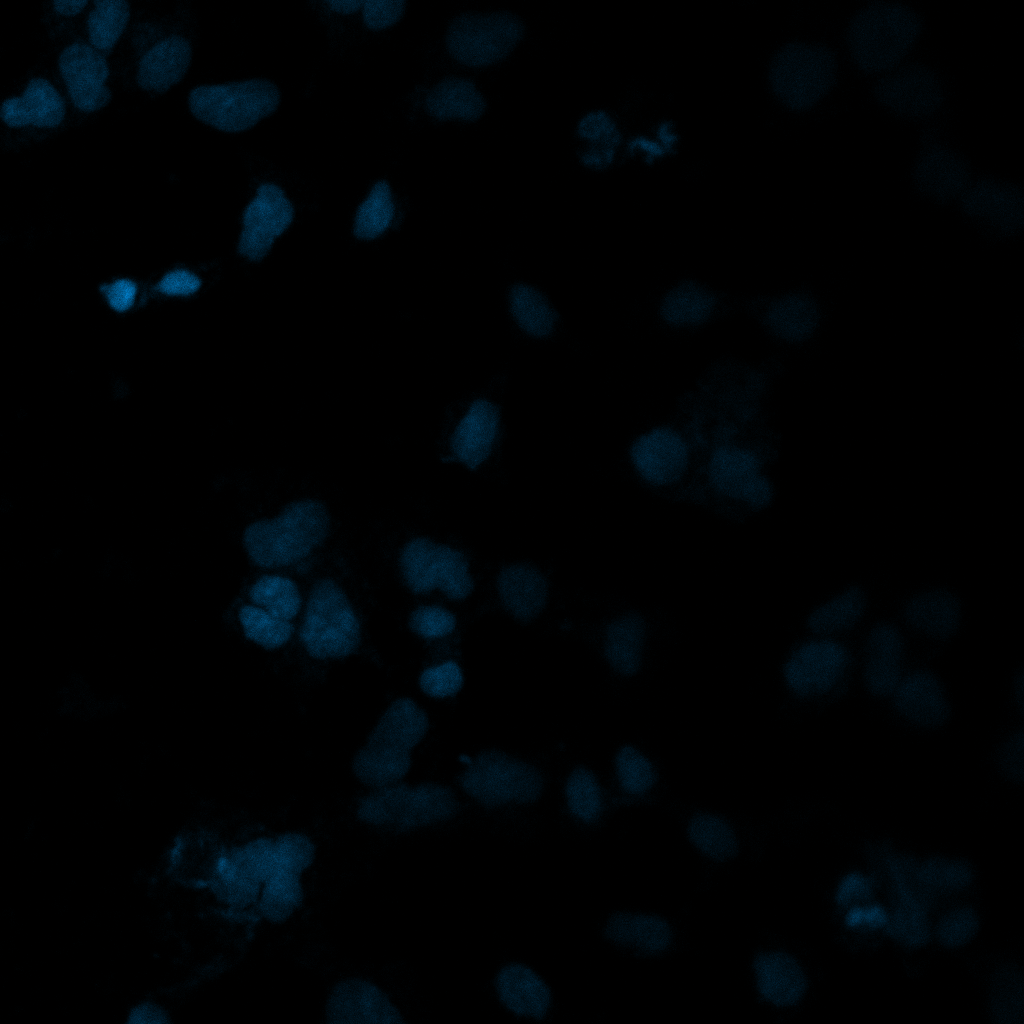

Supplement: Supplementary file 11 — Appendix Figure Source Data [file 44318_2025_636_MOESM11_ESM.zip › Figure S7/Figure S7B/Figure S7B-HEK293-DAPI.tif]

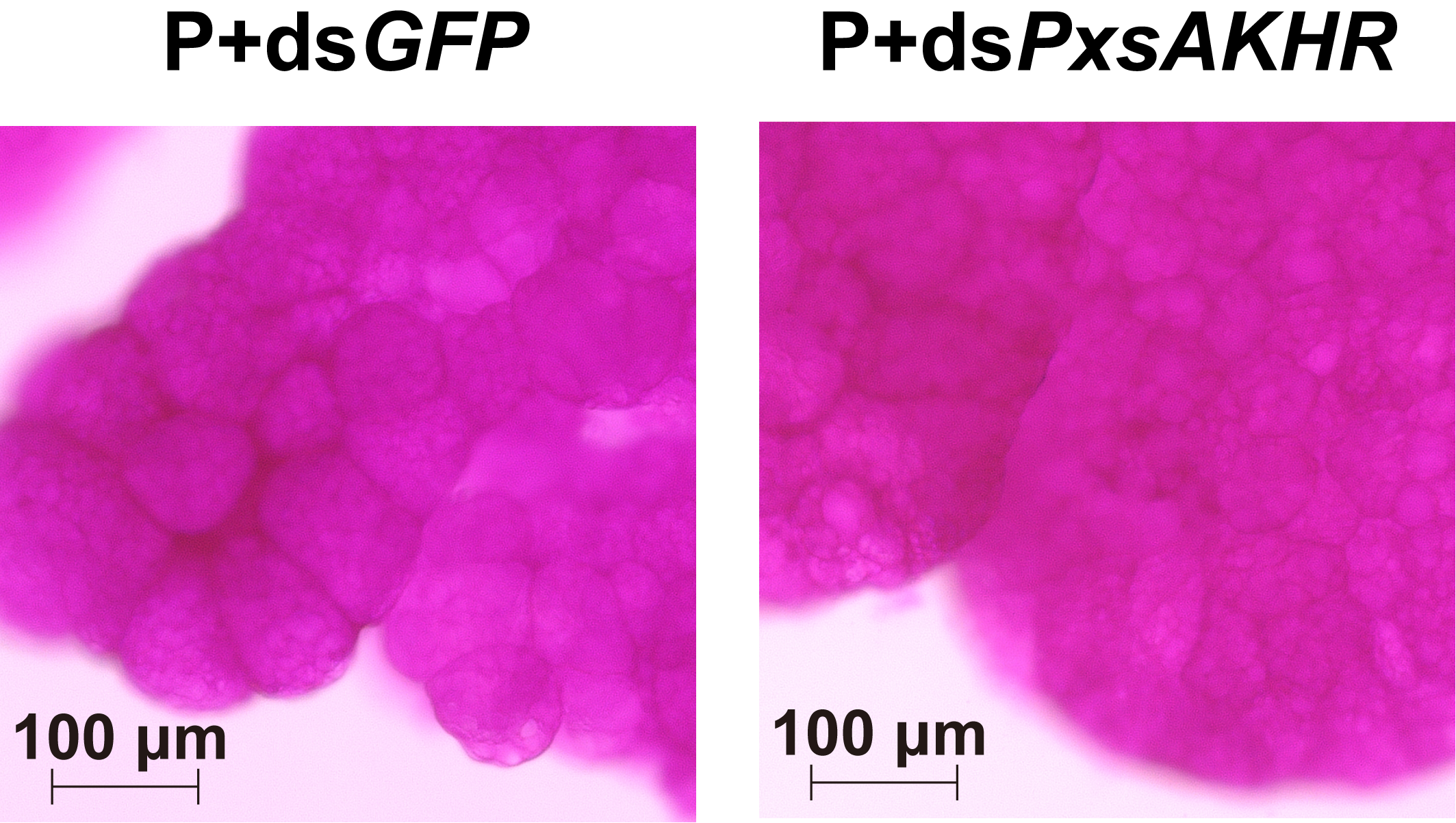

Supplement: Supplementary file 11 — Appendix Figure Source Data [file 44318_2025_636_MOESM11_ESM.zip › Figure S3/Figure S3C/Figure S3C.tif]

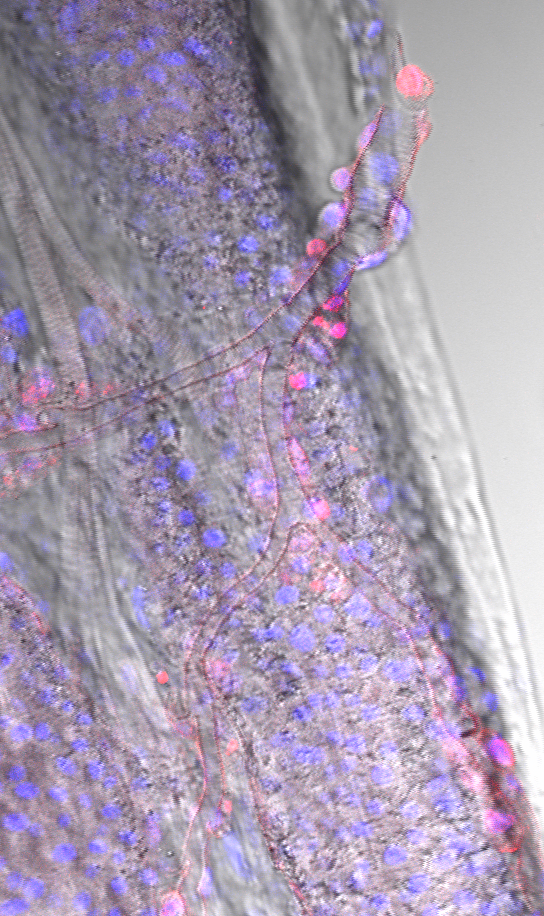

Supplement: Supplementary file 11 — Appendix Figure Source Data [file 44318_2025_636_MOESM11_ESM.zip › Figure S4/Figure S4B/Figure S4b-Tracheae-P.tif]

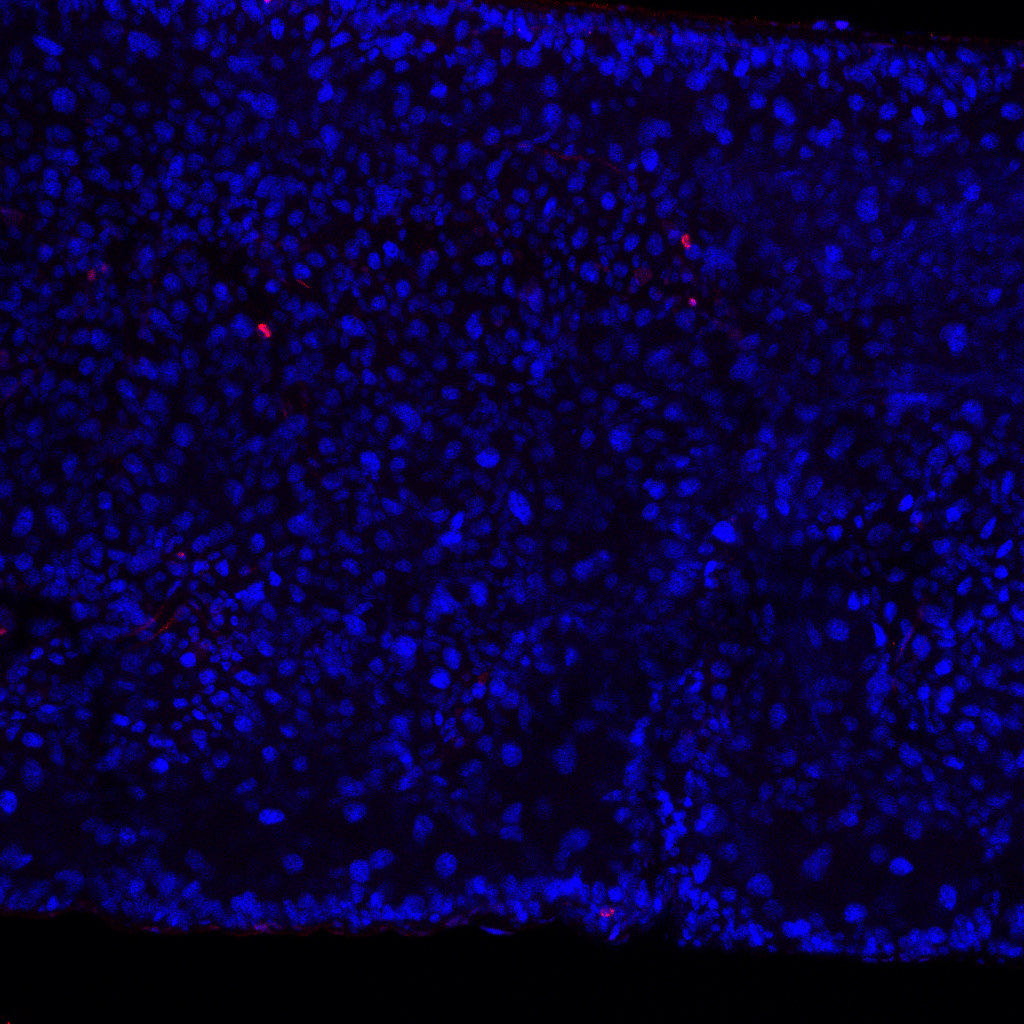

Supplement: Supplementary file 11 — Appendix Figure Source Data [file 44318_2025_636_MOESM11_ESM.zip › Figure S4/Figure S4B/Figure S4b-Intestine-P.tif]

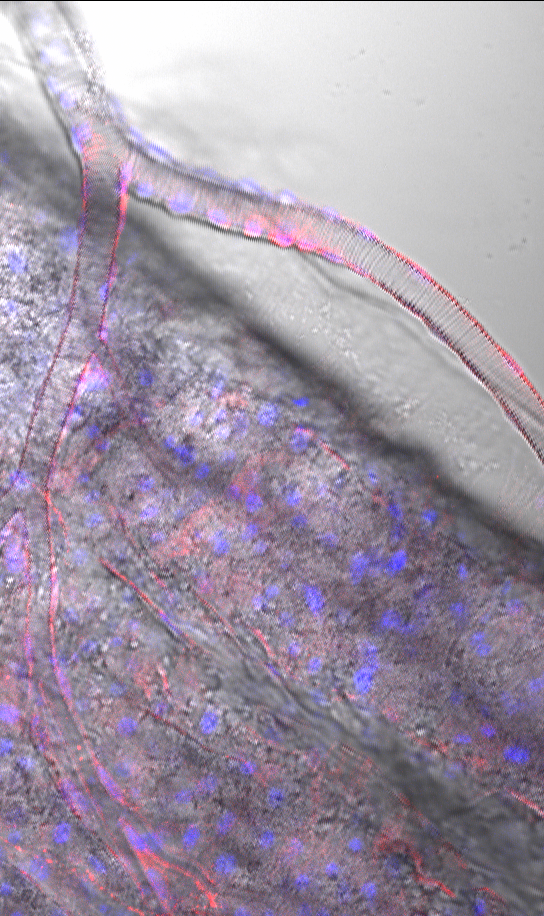

Supplement: Supplementary file 11 — Appendix Figure Source Data [file 44318_2025_636_MOESM11_ESM.zip › Figure S4/Figure S4B/Figure S4B-Tracheae-NP.tif]

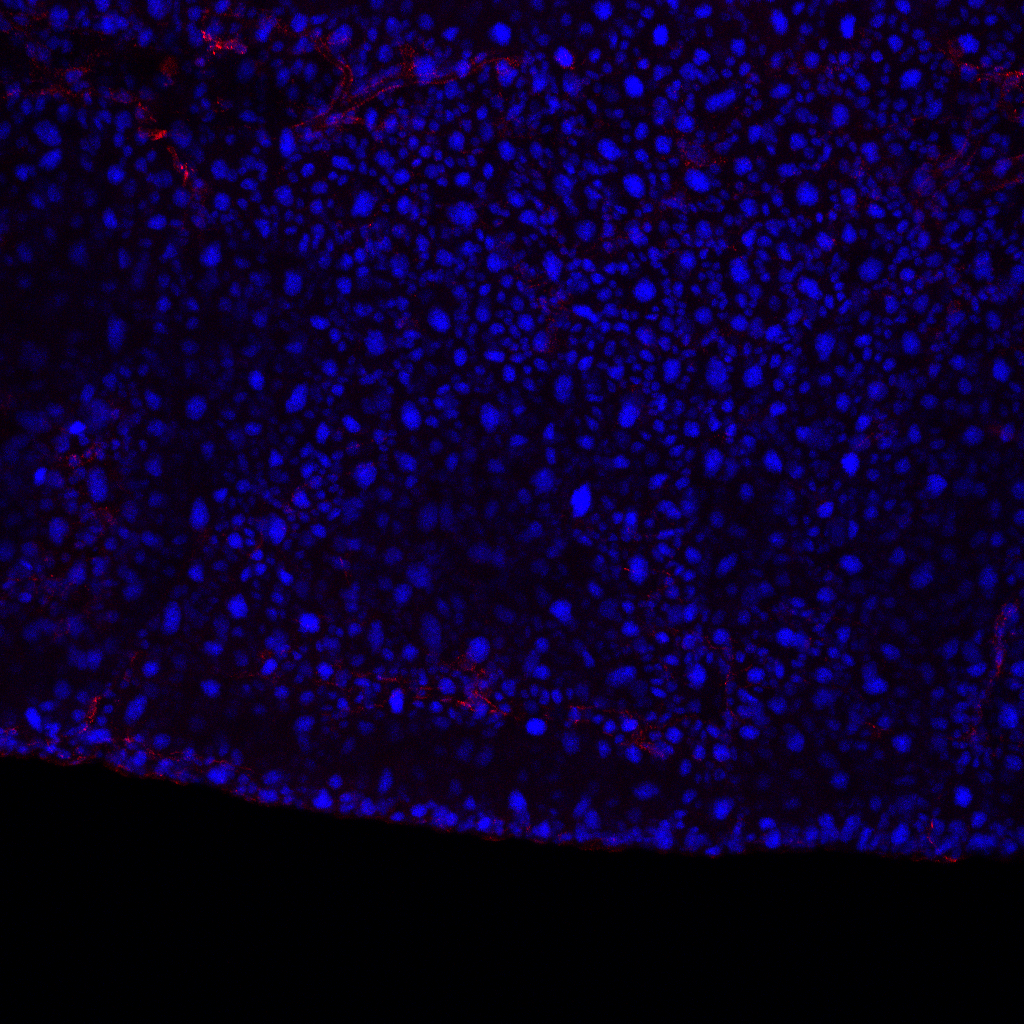

Supplement: Supplementary file 11 — Appendix Figure Source Data [file 44318_2025_636_MOESM11_ESM.zip › Figure S4/Figure S4B/Figure S4B-Intestine-NP.tif]

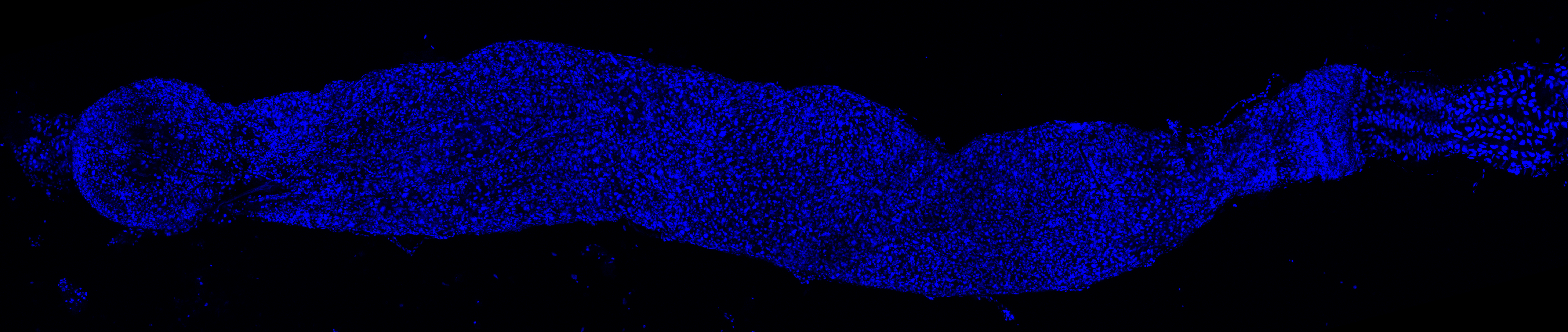

Supplement: Supplementary file 11 — Appendix Figure Source Data [file 44318_2025_636_MOESM11_ESM.zip › Figure S4/Figure S4C/Figure S4C-P-DAPI.tif]

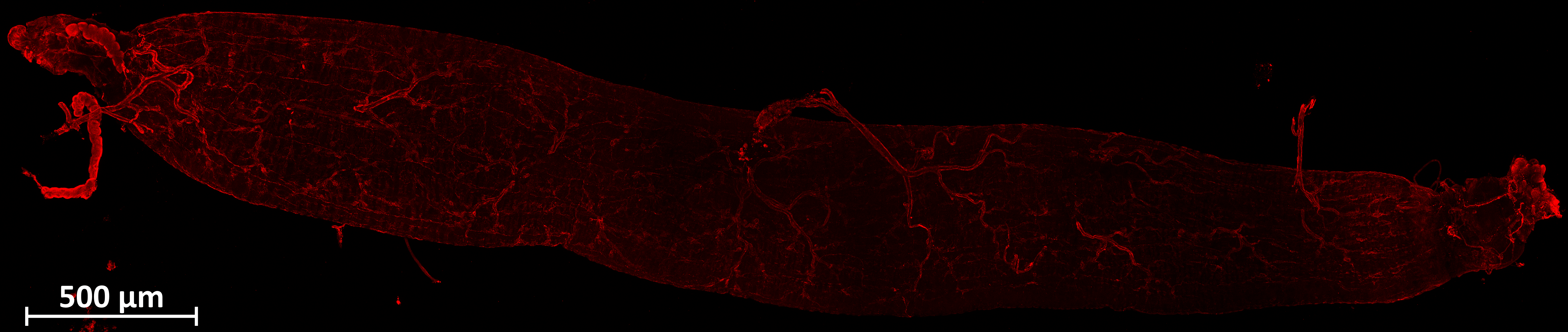

Supplement: Supplementary file 11 — Appendix Figure Source Data [file 44318_2025_636_MOESM11_ESM.zip › Figure S4/Figure S4C/Figure S4C-NP-sNPF.tif]

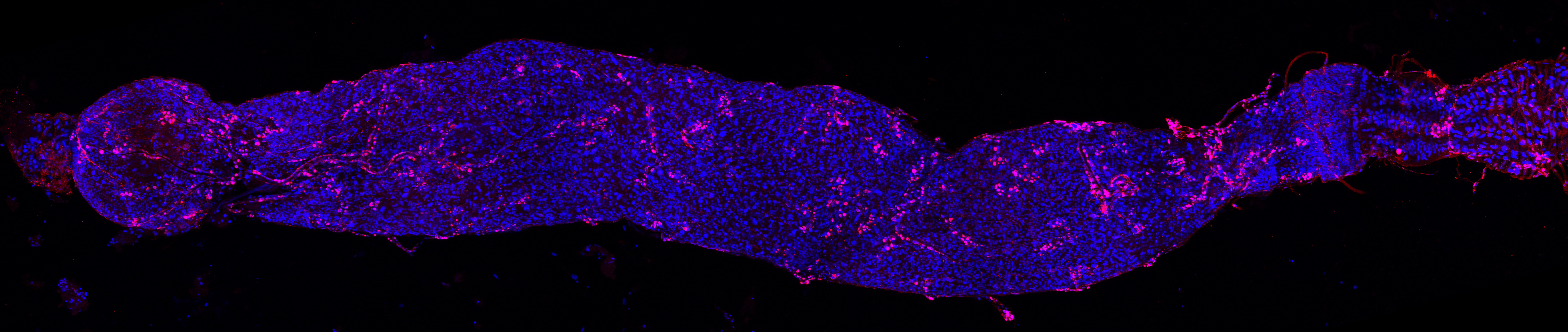

Supplement: Supplementary file 11 — Appendix Figure Source Data [file 44318_2025_636_MOESM11_ESM.zip › Figure S4/Figure S4C/Figure S4C-P-Merged.tif]

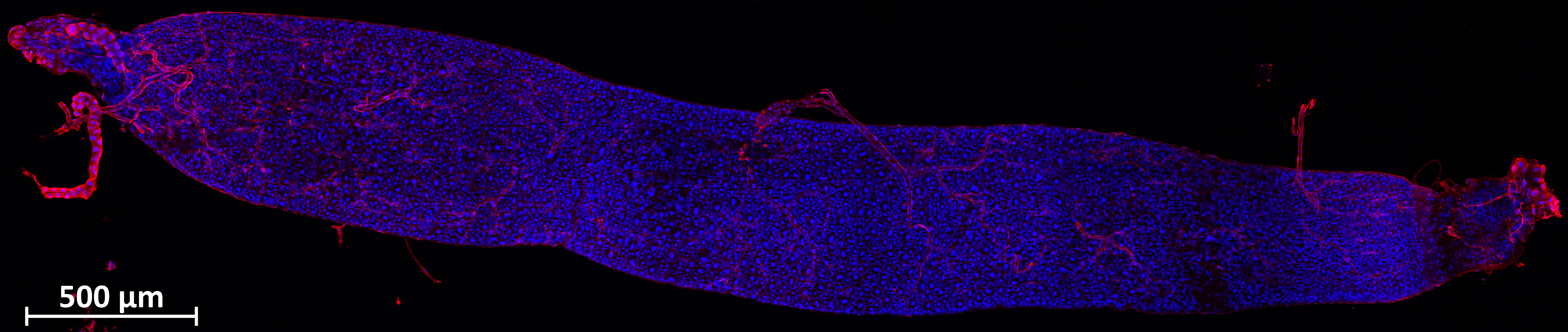

Supplement: Supplementary file 11 — Appendix Figure Source Data [file 44318_2025_636_MOESM11_ESM.zip › Figure S4/Figure S4C/Figure S4C-NP-Merged.tif]

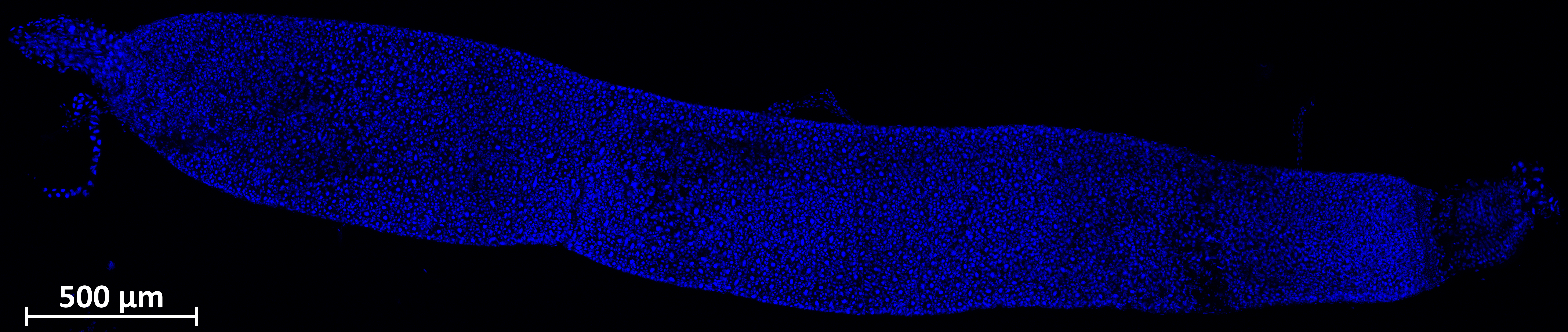

Supplement: Supplementary file 11 — Appendix Figure Source Data [file 44318_2025_636_MOESM11_ESM.zip › Figure S4/Figure S4C/Figure S4C-NP-DAPI.tif]

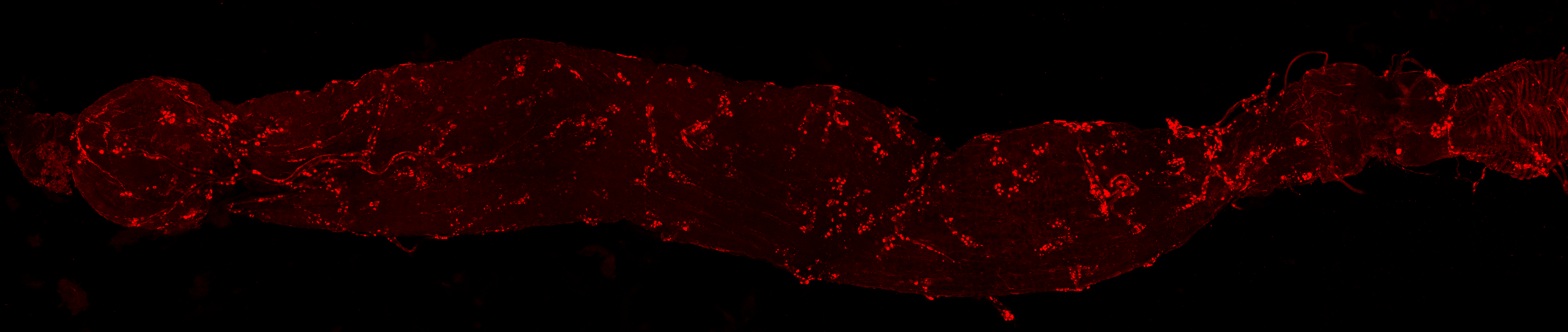

Supplement: Supplementary file 11 — Appendix Figure Source Data [file 44318_2025_636_MOESM11_ESM.zip › Figure S4/Figure S4C/Figure S4C-P-sNPF.tif]

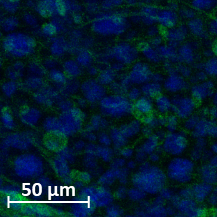

Supplement: Supplementary file 11 — Appendix Figure Source Data [file 44318_2025_636_MOESM11_ESM.zip › Figure S6/Figure S6B/Figure S6B-Intestine.tif]

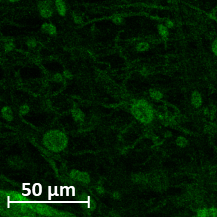

Supplement: Supplementary file 11 — Appendix Figure Source Data [file 44318_2025_636_MOESM11_ESM.zip › Figure S6/Figure S6B/Figure S6B-Intestine-PxsNPFR.tif]

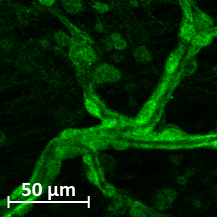

Supplement: Supplementary file 11 — Appendix Figure Source Data [file 44318_2025_636_MOESM11_ESM.zip › Figure S6/Figure S6B/Figure S6B-Tracheae-PxsNPFR.tif]

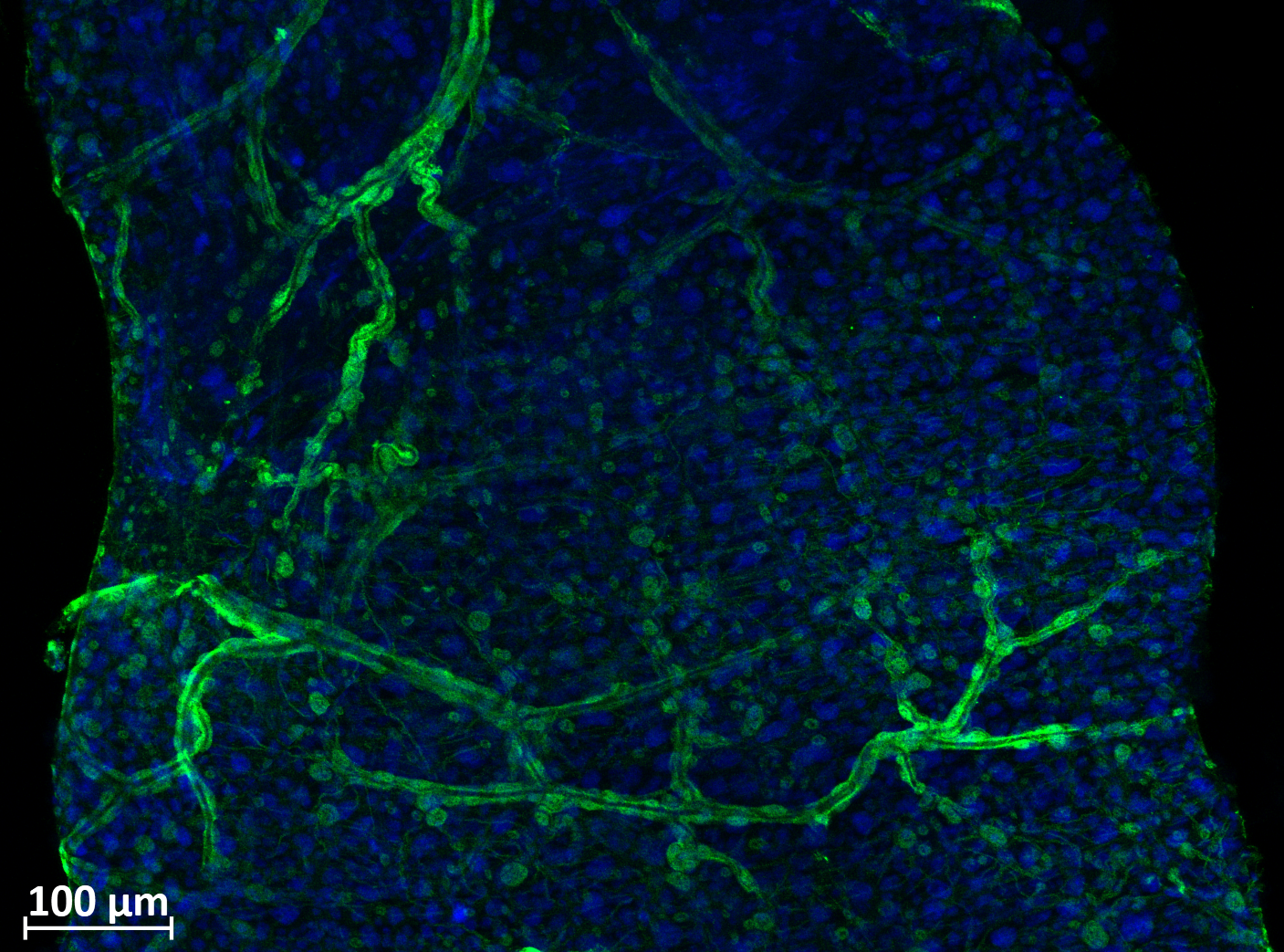

Supplement: Supplementary file 11 — Appendix Figure Source Data [file 44318_2025_636_MOESM11_ESM.zip › Figure S6/Figure S6B/Figure S6B-Midgut.tif]

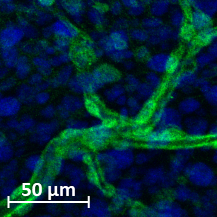

Supplement: Supplementary file 11 — Appendix Figure Source Data [file 44318_2025_636_MOESM11_ESM.zip › Figure S6/Figure S6B/Figure S6B-Tracheae-merged.tif]
